# Covid-19 Impact on the Music Sector in Belo Horizonte (Minas Gerais, Brazil)

**DOI:** 10.3389/fsoc.2021.643344

**Published:** 2021-06-29

**Authors:** Nísio Teixeira, Graziela Mello Vianna, Ricardo Lima, Carlos Jáuregui, Lucianna Furtado, Thiago Pereira Alberto, Rafael Medeiros

**Affiliations:** ^1^ Department of Social Communication, Laboratory Escutas, Fafich Federal University of Minas Gerais, Belo Horizonte, Brazil; ^2^ Department of Social Communication, Laboratory Escutas, Faculdades Promove, Belo Horizonte, Brazil; ^3^ Department of Journalism, Laboratory Escutas, Federal University of Ouro Preto, Mariana, Brazil; ^4^ Laboratory Escutas, PhD student at Federal University of Minas Gerais, Belo Horizonte, Brazil; ^5^ Laboratory Escutas, PhD student at Fluminense Federal University, Niterói, Brazil; ^6^ Laboratory Escutas, PhD student at Federal University of Santa Maria, Santa Maria, Brazil

**Keywords:** Belo Horizonte, Brazil, Covid-19, Music, social impact

## Abstract

This article addresses the difficulties of musicians and measures taken by public and private authorities to mitigate the social impact of Covid-19 in the music sector of Belo Horizonte, capital city of the state of Minas Gerais, Brazil. These are preliminary results of a research developed by the research lab on Sound, Communication, Textualities and Sociability [ESCUTAS (in Portuguese)] at the Social Communication Department of the Federal University of Minas Gerais. This study has two perspectives. First, we surveyed public sources about Brazilian measures for the sector, as we are interested in verifying policies used by the private and public sectors, not only at the national level, but also at regional (state of Minas Gerais) and local (city of Belo Horizonte) levels. Second, we investigate the impact of the pandemic on the city's music sector, considering various categories of the profession such as composers, interpreters, arrangers, music teachers, DJs, among others. This work is part of a scenario of academic research and economic reports on the impacts of the pandemic in the music industry. More specifically, it aims to contribute to discussion on the effects of the social distance on livelihood of professionals of that area.

## Introduction

In the end of October2020^1^ Brazil had surpassed the tragic mark of 157,000 deaths by Covid-19, and became the second largest country with victims of the new coronavirus, after the United States. The Brazilian state of Minas Gerais and its capital city, Belo Horizonte (BH), had their highest lethal incidence rate in the pandemic in the months of July and August. Despite a successful strategy of social isolation in March and April, the state had over 8,700 deaths, including over 1,450 deaths in Belo Horizonte^2^. This article presents a momentary overview of some of the most evident effects of pandemic times on the music sector in Belo Horizonte, based on the suggested outline. We show that a wide range of professionals in the field has been affected by the context of everyday life with Covid-19. In addition to deepening the inequality that already exists between categories, such as the drastic reduction in income by black professionals, the impacts of the pandemic also signal the increase in precariousness, already historically forgotten, in the universe of musicians outside the mainstream, with difficulty in establishing new sources income from their musical knowledge, in addition to the migration of musicians to other professional areas. In general, in view of this scenario, there is an urgent need for greater implementation of financial support initiatives by public and private institutions in order to establish commitments to assist this category during this period of crisis.

Belo Horizonte is the sixth most populous city in Brazil according to the last census (year 2010: about 2.3 million inhabitants). Inspired by the urban model of Paris and Washington D.C, Belo Horizonte was the first planned capital of the Brazilian Republican Age (proclaimed in 1889) being officially inaugurated on December 12, 1897. A modern design city installed, otherwise, in the heart of the mountains and the traditional society of Minas Gerais, which is illustrated by the Contorno avenue itself, which embraces the new city and was designated to receive the bureaucracy officials to the new capital, but excluded other important agents from its construction, such as bricklayers, railroad workers, weavers, who then began to occupy the neighborhoods bordering the main avenue (Aguiar Tito Flavio, 2018; Paula, 2018, Paula, 2020).

The former capital of Minas Gerais’s state, Ouro Preto was considered old, imperial and a place of the past, while Belo Horizonte is the new one, republican, and the place for the future. In the 1940’s the mayor and Brazil future president Juscelino Kubistchek invite Oscar Niemeyer to continue the modernization project of the city, especially on the new frontier of town, the Pampulha area–but still a modernization loss with traditional problems as a lack of social policies faced a continuous and disorderly population growth–that persists into the present day (Barreto, 1944; Chacham, 1994; Bahia, 2004; Bahia, 2011; Brandão, 2018; Costa, 2019; Amormino, 2020). This tension between the modern and traditional in Belo Horizonte is not only express by history of the city itself, but also for several of the artists that lived in the city, from Brazilian writers and poets known worldwide like João Guimarães Rosa, Fernando Sabino and Carlos Drummond de Andrade to performing artists like the Galpão theater group, Corpo dance group, Giramundo puppet theater group and–now finished–Uakti music group. All of these groups, ironically, were created during the Winter Cultural Festivals promoted by Federal University of Minas Gerais in the city of Ouro Preto (Dias, 1975; Werneck, 1992; Paula, 2020). The musical scenario in Belo Horizonte is also internationally known due to artists like Milton Nascimento, Toninho Horta, Lô Borges, Beto Guedes, Fernando Brant and other names connected to the important musical movement called Clube da Esquina (Corner Club) forged on the streets and corners of Belo Horizonte in the 1970’s, while in the 1980’s the band Sepultura, which grows in the traditional streets of Santa Tereza neighborhood, gained the world with their heavy metal style. (Dolabela, 1987; Teixeira, 1994, Teixeira, 1996; Dolabela, 1997; Martins, 2009; Grasse, 2020). The musical scenario in Belo Horizonte has great diversity, creative quality, innovation and growing dynamism, and all of these ingredients were considered to propose a Local Production and Innovation System (SPIL by short) (Medeiros and Machado 2015) or even a local system of information and/or cultural incubators, for example, to rock bands (Teixeira, 1999, Teixeira, 2008).

On October 31, 2019, the city was awarded the title of Creative City by UNESCO for its gastronomy, a sector that is fundamental for the so-called “bar capital of Brazil,” or a city where a popular quote (that rhymes in Portuguese), “there’s no sea, let’s go to the bar,” makes total sense; so much so that the New York Times has mentioned it twice in articles about the city (Kugel, 2007, Kugel, 2014). Therefore, in a good part of the estimated 12,000 bars and restaurants in the city, musicians find an important place to increase their income, since their presence in these spaces is usual and frequently requested, although far from being fully and properly adjusted to their demands.

The city is also internationally known for the excellent convergence of Brazilian and Minas Gerais cuisine, as well as for its musical diversity, but the expectation of a favorable 2020 and a consequent improvement of the sector was frustrated given the impact of Covid-19. With the closure of bars, nightclubs, concert halls and other cultural spaces, musical performances were suspended and, in the case of Belo Horizonte, a gradual and restricted return only began on September 4th, 2020, with very restrictive measures for musicians to return to bars and restaurants after September 19th, 2020^3^.

Despite the relevance already demonstrated of making music in Belo Horizonte and the importance of a research like this and its intention to capture some local conditions of this profession in the pandemic context, we should highlight our comprehension here of music as “musicking”: A place where countless interactions are dynamized that, in fact, provide the meaning of musical material, as it is perceived, experienced, lived (Small, 1998). Still, we consider music in its role of mediator between actors which justifies an investigation that seeks the social in art, pointing out devices triggered by music to exist, such as singers, instrumentalists, sound systems, sound, spaces for musical performance, forms of sound recordings, producers, consumers, dancers, microphones etc. Such understanding allows us to direct the investigative look into a perspective of relations that these professionals establish between themselves and their practices into socio-cultural-process, and conditions involved in this very “local musicking” (Hennion, 1993; Campos, 2007). We should look for indexes of the way the agents practice and relate to musiking in their respective contexts, times and regimes of collective association, and how music emerged in their everyday life, guiding the daily lives of individuals, interfering in their experience of time, impacting the way people understand themselves and in relation to the collectivity to which they belong. (Weber, 1998; Denora, 2000). It means to say that art is constituted as a pleiad, as a cosmos of conscious, comprehensive, and autonomous intrinsic values ​​capable of revealing itself as a privileged object to think about social dynamics (Habermas, 1984). Music, then, is intrinsically instituted as a production of the sensitive spirit, but which gains significance, including esthetics, insofar as we can perceive it in friction with the historical, political and social context with which it dialogues (Wisnik, 1989).

The impact of Covid-19 at the present moment (and even when it is less serious) will certainly focus primarily on the fields of health and education, in order to guarantee the proper functioning and protection of all professional bodies involved directly or indirectly in these sectors. However, a Reuters survey promoted by the Ipsos News Agency and Institute published in late April showed that, in face of the Covid-19 pandemic, only 27% of Americans admitted that they would return to attending concerts, cinemas and theaters when they are authorized to reopen. 32% said that they would only return to these places when there is a vaccine (Carrol, 2020). Based on this premise, the roles of culture, sports, art and entertainment, which have also been important in supporting not only the physical and also the mental health of the population throughout this moment of predominant domestic permanence, but also should therefore be recognized for their key role on economic recovery plans, even if only proportionally.

The social distancing necessary to reduce the spread of the new coronavirus highlights the importance of music in order to minimize anguish and the worsening of the symptoms of different mental conditions (depression, anxiety etc.), even though we are temporarily prevented from sharing the same physical space of the musical enjoyment experience. This perception is emphasized by the *Music Cities - Resilience Handbook*, available at #BetterMusicCities and produced by specialists from Sounddiplomacy.com, who work with music economy for urban development (Sound Diplomacy, 2020). At the beginning of the pandemic, this collective publication warned about the impact that the music industry would suffer, and listed reasons why cities should include support for music professionals in their planning, as well as action strategies. In this sense the commotion caused by the pandemic could catalyze a structural change in the management of the cities, which was a need that had been made invisible:

For decades, sport has successfully made the case that it delivers unarguable returns on investment in terms of public health and wellbeing. But music has never quite managed to make its case in that arena. Now, in the most bleak moments of this current crisis, we see clearly the need, the impact and the results of music and culture in delivering positive outcomes in both physical and mental health. Music has demonstrated the power and benefits of social prescribing like never before (Sound Diplomacy, 2020).

In this scenario, during the pandemic, a lot of Brazilian musicians recorded free of charge videos or broadcast live performances (the so-called “lives”). Brazil even reached an audience of about 85 million people watching “lives,” according to data released by Google in August 2020, which places the country as the world leader in the audience of “lives” at that time^4^ (Amaral 2020). But despite this high audience rate, only a little few, among these musicians, obtain any type of payment–either from public or private sponsorships or by the monetization of platforms, as some of the results of this research will show.

The passing of the Aldir Blanc law (created in honor of the important Brazilian composer victim of the coronavirus on May 4, 2020) in the House of Representatives and the Federal Senate provides an aid of R$ (BRL) 3.6 billion (US $ 641 million)^5^ for the cultural industry. This law was sanctioned on August 13, 2020. Despite these apparently high numbers (considering the Brazilian economic reality), this amount attenuates only about one third of the impact of the pandemic on the Brazilian artistic sector: a technical note released on April 30, 2020, by the team of researchers from the Regional Planning and Development Center (Cedeplar, in Portuguese) from the School of Administration and Economic Sciences of the Federal University of Minas Gerais (Face/UFMG in Portuguese) stated that “The impact of the interruption of the provision of artistic and cultural services outside the home for three months would represent a drop of R$ 11, 1 billion (US $ 1.977 billion) in the production value of the Brazilian economy”—which would represent a reduction of about one fifth of the gross production value of the sector itself in the year (Machado et al., 2020). So, the great paradox in the COVID-19 era is that the public is consuming art, music and cultural goods at an accelerated rate, but they are not contributing enough financially to ensure that the artists, musicians, and creatives whose work they are consuming can meet their own basic needs. For the vast majority, their art is not paying the bills, nor are the second and sometimes third jobs they have to take, just to retain their ability to create (Sound Diplomacy, 2020, 11).

Belo Horizonte, like other cities, has also received several initiatives and actions such as public calls for applications and projects from public, private and third sector institutions–at federal, state and municipal levels. These programs have been directed to the artistic class in general, and to the musical class in particular. Several of these actions have been compiled and analyzed by the members of this research group and a review of these support initiatives will be presented later in this paper.

This work is part of a scenario of academic research and economic reports on the impacts of the pandemic on the music industry. More specifically, it aims to contribute to discussion on the effects of the social distance on livelihood of profissionais of that area. At this right moment, it is considerably difficult to make a panorama of those studies, since it is a very specific field and is still in formation. But we understand the importance of identifying and mapping both those works developed in the context of developed countries (Betzler et al., 2020; Dones et al., 2020; Hunt et al., 2020; Tolmie 2020; Crosby and McKenzie, 2021; Spiro et al., 2021) and those who look at developing economies (Joffe 2020; Varano 2020).

As we see below, this research was approved by the Department of Social Communication Chamber and also by the Research Ethics Committee of the Federal University of Minas Gerais. With these results, this study of ESCUTAS Research Group^6^ makes visible the difficulties faced by the music sector in the context of the Covid-19 pandemic and the significant role and importance of this sector on several health and economics levels in society.

## Methods

### Research Tools

In order to systematize the research data questions, as mentioned above, we carried out a preliminary review of the actions taken by several cities around the world to mitigate the impact of Covid-19 on the cultural sector, before focusing in particular on the capital city of Minas Gerais.

We started the world panorama with a list that was available on March 30th, 2020 at Cités et Gouvernements Locaux Unis (CGLU) website about the cultural mobilization in several cities and local governments due to the COVID-19 crisis (Cités et Gouvernements Locaux Unis, 2020). And throughout the month of May 2020, we discussed on virtual meetings the cases availables of the first political actions taken from some cities from America, Europe and Asia: Berlin (Alex Berlin, 2020; Kulturleben Berlin, 2020; Berlin - Senate Department for Culture, 2020; Berlin(A)Live, 2020; Berlin Buehnen, 2020; Berliner Philharmoniker Digital Concert Hall, 2020; Berlin - United We Stream, 2020); Boston (Carroll, 2020a, Carroll, 2020b; Boston Singers Resource, 2020); Hong Kong (Art Power, 2020; Hong Kong Arts Development Council, 2020); Lisbon (Lisboa, 2020); Montevideo (Montevideo - Cultura, 2020a, Montevideo - Cultura, 2020b; Montevideo, 2020; Noceti, 2020); New York (Americans for the Arts 2020a, Americans for the Arts, 2020b; NYC Cultural Affairs, 2020; Peoples Cultural Plan 2020a, Peoples Cultural Plan, 2020b) and Paris (Ville de Paris, 2020). Meanwhile, we attended a call to integrate the #Musicovid Network, an international research network which had an international meeting on May 19th, 2020 which gathered more than 80 ongoing studies and surveys around the world (Musicovid Network, 2020).

So, after this preliminary research contact about similar subjects in other cities, some questions came up and motivated our own research: How do the musical professionals–whose income depended mostly on face-to-face activities–survive in pandemic times? What initiatives are being taken to ensure such survival? These questions lead to the main results of this paper: a review on public and private policies for the sector, and a survey into the Belo Horizonte music industry.

The second research method step was a categorization of the social actors related to this musical sector in Belo Horizonte–understanding that this categorization does not exhaust the entire network of actors who work in the area. Our preliminary categories are: 1) Music professionals; 2) Public and private institutions and their actions towards the musical sector and 3) Managers or owners of show/entertainment places. We consider to refine this categorization in further phases of the research.

For the first category we developed a survey as a research tool to investigate the music professionals. Surveys are one of the most commonly used methods in social sciences to understand many social and cultural aspects of the communities (Groves et al., 2009). As we said, the elaboration of this survey was inspired by precedent research about how the impact of Covid-19 affected other cities, especially the survey applied about this impact in the United States (Americans for the Arts, 2020a; Americans for the Arts, 2020b) We developed our own research tool inspired by those studies and, of course, considering the social, cultural, political and economic contexts of Belo Horizonte. The music professionals will be also investigated in a focus group stage to get deeper into some questions that we are interested in and to generate qualitative data (Morgan, 1993; Krueguer and Casey, 2014) in the second stage of this research.

In the second category–for public and private initiatives–the research tool was documentary research based on collected information in publicly accessible web sources, not only those of merely institutional character but also news websites, cultural revues and others. Finally, even though we also developed a first draft of a specific survey for managers or owners of show/entertainment venues, we decided to investigate this third category in a further study due to practical and operational reasons, and, of course, due to the wide research arc required from the two stages mentioned before.

### Research Procedures

The present research was produced considering characteristics of the snowball sampling method (Biernarcki and Waldorf, 1981; Bernard, 2005) especially for two reasons: first, the presence of sensitive information, such as the income range of the musician before and, above all, after the context of the pandemic in which the financial difficulty and the increased anxiety and loneliness of the artists became evident (Spiro et al., 2021). Then, due to the very context of social isolation to which the whole city of Belo Horizonte was subjected during the research period, a direct collection of an initial sample became simply impractical. The snowball sampling also came as a solution as we may see in other works and areas under the context of Covid-19 (Ali et al., 2020; Hanage et al., 2020).

This method was used also for exploratory purposes, to better understand the scenario and finally to test the possibility of a broader and subsequent study. Since its first page, the survey makes clear for the interviewer their objective and desired profile. So each of the authors submitted to different respondents, who became multiplier seeds for other respondents in a personal chain of contacts that generates reliability for the interviewer (Becker, 1993; Vinuto, 2014).

The quantitative and qualitative survey consisted of applying Google Forms questionnaires with open-ended and closed-ended questions to professionals in the music sector whose main activity is one of the following: composers, interpreters, arrangers, conductors, DJs, technicians, music directors and executives, producers, music teachers and luthiers. This categorization was defined after the review of international city cases mentioned above plus previous work from the ESCUTAS researchers on the musical scene in Belo Horizonte (Teixeira, 1999).

### Sample

The survey was disseminated through the researchers networks, with the support of the University’s internal communication vehicles (radio and website^7^), and also the regional and local press (TV; radio stations; print and web media^8^). The answers we have analyzed in this article were obtained from August 10 to October 6, 2020. 174 professionals from these categories answered the questionnaire, and the main results of this survey will be also presented in the next section of this paper^9^. As mentioned before, in a further stage of this research, focus group meetings will be held with at least two representatives from each function specified above, who have expressed interest in participating in the next phase of the study. These representatives–at least 20 participants–will be chosen from the survey final open-ended answers.

As for the age group aspect, from the total number of valid responses 36 were from people between ages 18–30, 54 between ages 31–40, 46 between ages 41–50, and 35 age 51 or older. The distribution of respondents by gender is as follows: 135 male, 33 female, one non-binary and two people preferred not to respond. As for education level, 9 people have incomplete high school; 23 have complete high school; 35 have incomplete university education; 59 have complete university education, 6 have incomplete post graduation and 39 have completed the post graduation level. In terms of racial identity, from a total of 171 respondents: 100 self-declared whites; 64 blacks (the sum of dark skin and light skin Black people, according to the IBGE criteria), one self-declared as yellow, no one identified as indigenous and 6 chose not to respond. As for their professional practice in the music community, we started from the premise that many of the respondents have different profiles and conciliate more than one area of ​​activity, whether close and complementary or not, as will be seen below. So we gave them the possibility to choose more than one answer option. Therefore, we identified that many (141 responses, 82.5% of the total) choose more than one of the options offered. Only 30 (17.5%) of the respondents announced a single work area, among the following categories: composition; interpretation (voice); interpretation (musical instrument); arrangement; conducting; production (executive), production (musical), technical aspects, teaching, research; DJ and luthier. The answers tell us that the areas of expertize with the greatest involvement by the professionals interviewed were: interpretation (musical instrument) with 111 respondents; composition, with 83; interpretation (voice), with 80; musical production with 67; teaching, with 60; executive production with 52; arrangement with 50; art direction with 41; technology with 30; DJ with 23; research with 19; conductor with 11; and luthier with 8 respondents.

Therefore, we have a group that is mostly formed by interpreters (voice and/or instrument) who are white, male, older and highly educated. Although the aim at this stage of our research is to present the survey’s preliminary results, considering the participants characteristics in terms of privilege and social status, we found it important to examine the pandemic’s impact on their income in relation to their racial identity (see *General considerations on income and social and racial inequalities*). This is due to the possibility that this data will ground the development of public policies for the music business and, more broadly, the cultural sector’s economic recovery when the city reaches a significant vaccination rate, hopefully taking affirmative action in consideration in order to promote racial equality. In our future work, we intend to further develop the analysis of our data considering different variables such as gender and age group, as well as the workers' specific professions in the music sector and their additional occupations.

### Ethical Issues

The questionnaire sent to music professionals as described in the research procedures has a Free and Clarified Consent Term (“Termo de Consentimento Livre e Esclarecido” - TCLE in portuguese) where the respondents were invited to voluntarily participate in the research. The survey also informs in its consent “that the collected data will be used only for the research. At any time, whether before, during or after the survey, the respondent could request further clarification, refuse to participate or withdraw from participation. In any of these cases, he would not be harmed or held responsible in any way”. (*Survey is available in the appendix*).

During the months of June and July 2020, before sending the survey to potential respondents, we submitted the proposal–including the role research project, the survey and the term of consent (TCLE)—to the Ethics in Research Commitée (CEP) linked to the National Ethic Commitée System (CONEP). These Comitées are responsible for ethical evaluation in research protocols involving human beings to protect the well-being of individuals participating in research carried out in the universities (COEP, 2021; CONEP, 2021). The CONEP gathers researchers in different levels (undergraduate, PhD researchers, professors, etc). The rigor of evaluations is known across the country. Finally, the research projected and survey was approved by the Department of Social Communication Chamber and also by the Research Ethics Committee of the Federal University of Minas Gerais^10^.

## Results

The results presented in this paper were discussed by the ESCUTAS research group in biweekly online meetings, daily emails and whatsapp messages and a collective writing platform on Google Drive, in October and November 2020. They considered the first and second categories listed above and had the two following parts: a review on public and private policies created to mitigate the Covid-19 impact on the cultural sector, and a survey analysis. In a future stage of this research, the survey answers will be compared with the analysis of focus group results. Then the results will be gathered in order to elaborate a broad diagnosis of the musical environment in Belo Horizonte, to be published and sent to all the survey respondents.

### Documentary Research About Public and Private Policies for the Sector: Preliminary Results

In the documentary research, we made the effort of gathering together the spread information about policies created by public and private institutions to mitigate the impact of Covid-19 for the cultural sector, which results we present in this section of the paper. We believe these results can highlight the local contexts where the music professionals are immersed.

Public policies to assist workers in the cultural sector during the context of pandemia in 2020 have been structured in all three spheres of Brazilian public administration–federal, state and municipal. Institutions participating in each of these spheres have allocated specific projects and public calls for application to professionals in the cultural area, in order to mitigate the impact of artistic presentations being cancelled and the closing of concert halls and cultural centers. These policies have been mapped in the present research in order to understand, in sync with the questionnaire responses, the extent to which they are able to lighten the difficulties faced by professionals and groups in the cultural sector.

At the federal level, the Brazilian government’s performance in the context of the pandemic in relation to culture was classified as very slow (Rodrigues 2020), since only two actions were taken, seven months after the declaration of the world pandemic state by the World Health Organization (WHO) in March 2020. The most comprehensive public policy on a national scale is the Aldir Blanc Cultural Emergency Law, enacted on June 29, 2020, but whose practical effects started in September 2020. Approved after political and artistic pressure, the law seeks to give institutional support to professionals in the cultural sector (Brant and Fernandes 2020) and provides the transfer of R$ 3 billion (US$ 534 million) from the National Culture Fund to be applied by states and municipalities in emergency actions for the cultural sector during the pandemic.

The payment of a basic monthly income of R$ 600 (US$ 107) to workers who can prove that they worked in the cultural sector for the last 2 years is one of the actions that directly impact professionals in the category. To have access to this aid, the artist cannot have a formal employment relationship and cannot receive other assistance or social security benefits, unemployment insurance or values ​​from federal income transfer programs (except for the Bolsa Família^11^). According to a projection made by the Applied Economic Research Institute (Ipea, in Portuguese) based on data from the Brazilian Institute for Geography and Statistics (IBGE, idem), the number of citizens that benefit directly from the cultural emergency aid should vary between 400,000 and 700,000 workers at the national level (Góes et al., 2020).

In addition to professionals, the Aldir Blanc Law also includes artistic and cultural facilities, micro and small cultural companies, cooperatives, institutions and community cultural organizations. These institutions are entitled to a subsidy ranging from R$ 3,000 (US$ 534) to R$ 10,000 (US$ 1,700) and, in return, they must promote free cultural activities for students of public schools and their communities after the restart of activities. In general, the legislation is comprehensive in naming 24 types of cultural venues that can be supported by the grant, among which are independent theaters, music schools, film and audiovisual producers, spaces for musical presentation, circuses, libraries, popular celebrations, cultural places in indigenous communities and african-descendant cultural centers, in addition to other facilities and activities validated in the state and municipal cultural registries. Other actions foreseen by federal law are the issuance of public calls for applications and prizes to develop a creative and solidarity economy within the sector for promoting artistic and cultural activities that can be transmitted online, and the availability of credit lines for fostering cultural activities, purchase of equipment and debt renegotiation.

It is important to highlight that the decentralization of resources, transferred from the Federal Government to states and municipalities and managed preferably through culture funds, would theoretically imply a speedy allocation of funds and their efficient application, facilitated by the identification of local demands. However, due to lack of organization or political reasons, many municipalities are failing to map out the citizens who have the right to receive assistance and are not requesting the resources guaranteed by the legislation. According to data from the Ministry of Tourism, 26% of the 853 municipalities in the state of Minas Gerais have not yet sent an action plan to the federal government and are at risk of not getting the money, because when the municipality does not request the money allocated to it, the resource is transferred to the state culture funds (Maciel, 2020).

In addition to managing the resources of the Aldir Blanc Law, states and municipalities are also developing their own alternatives to assist the cultural sector in the context of the pandemic. Some actions of the Minas Gerais government in this area started to be taken in March 2020, when the Department of Culture and Tourism relaxed the rules for the execution of previously approved artistic projects in cultural incentive mechanisms that could be adapted to the digital format.

Another initiative at the state level was the opening of the Arte Salva public call for application in June 2020, which provides for the distribution of R$ 2.5 million (US$ 447,000) in prizes for independent artists, bands, circus professionals and other actors that make up the cultural scene of Minas Gerais. 1,315 projects will be awarded, and each will receive an investment of R$ 1,900 (US$ 338) for the realization and availability of pieces of artistic and cultural expression in a digital environment. This call, which is still in progress, accepts projects in different languages: music, performing arts, visual arts, audiovisual, heritage, literature and integrated cultural areas.

At the municipal level, the Municipality of Belo Horizonte initiated its actions with administrative procedures and reopened the registrations for the Municipal Cultural Incentive Law (a local modality of tax incentives for cultural events). It also temporarily suspended the receipt of accountability documentation for cultural projects, the execution of projects already approved and their respective social returns in order to reduce the bureaucracy of access to incentives.

As a form of direct financial support, the Belo Horizonte City Department of Culture also created “Circuito em Casa” (“Circuit at home”), an artistic-cultural festival with a schedule that includes live online broadcasts and exhibitions of videos, short and feature films, debates and training activities, musical attractions, performing arts, literature, audiovisual, visual arts and popular culture, with focus on local artistic production. The first part of the program is intended for events that had been previously planned and were suspended due to the pandemic. For the second part of the project, a public call was issued to register new proposals by artists, cultural agents and workers in the cultural production chain of Belo Horizonte and the Metropolitan Region. The curatorship will select 45 projects, with a forecast of R$ 1,000 (US$ 178) for each (Buzatti, 2020).

Public policies of other agencies can also be cited as relevant initiatives in the current situation. At the Belo Horizonte City Council, the Cultural Restart Bill, whose voting is still pending, foresees the construction of an action plan to enable the return of artistic and cultural activities in a safe and democratic way. In April, the Federal University of Minas Gerais (UFMG) promoted the Cultural Promotion Program, an initiative that offered R$ 900 (US$ 160) scholarships to students of the University to carry out unpublished and copyrighted artistic, essayistic or literary works about the implications of the pandemic. Proposals for productions in the areas of visual arts, photography, video, performance, dance, theater, circus, music, short stories, poetry and romance were accepted, as well as essays in all areas of knowledge (Universidade Federal de Minas Gerais, 2020).

It is also necessary to consider the emergency aid aimed at informal and self-employed workers among the government public policies that impact professionals in the cultural sector. It is the main economic support program for the population in the context of the pandemic and its beneficiaries include the unemployed, informal workers and individual micro-entrepreneurs, so it includes professional categories from the cultural sector. The so-called first cycle of the program, which started in April 2020, consisted in the payment of R$ 600 (US$ 107) or R$ 1,200 (US$ 214) (in the case of single mothers with children under 18 years) for three months (Cristaldo, 2020). In the second cycle, which started in September, the amounts were cut in half and were to be paid for four months. According to the federal government, 109 million registrations have been processed to date and 67.7 million people have benefited from the emergency aid (Vilela, 2020). The data released by government agencies do not allow to identify the exact number of workers in the cultural sector serviced by the program.

Institutes linked to private organizations, institutions of the so-called third sector and other non-governmental organizations are also developing projects for the cultural sector in this period. Emergency calls were launched by Itaú Cultural and Sesc, covering cultural activities from different segments including visual performances, production of short films, theatrical and musical presentations and storytelling. The two calls for application had an expressive number of subscribers (there were 12,396 candidates for the Itaú Cultural announcement dedicated to music alone) and the amounts offered for each selected proposal varied between R$ 1,000 (US$ 178) and R$ 5,000 (US$ 891). (Itaú Cultural, 2020).

Ethnic, racial and social minorities are the groups that are most vulnerable to the effects of the pandemic, considering their health condition but also considering their economic situation (Alves dos Santos et al., 2020). As will be shown below, the people who answered the questionnaire and identified themselves as black are predominantly in the lower income statements and suffered more from the earning decrease in the context of the pandemic. In this sense, specific support for cultural sector professionals belonging to these minority groups has been successful through associative initiatives, such as the Association of Black Audiovisual Professionals (APAN), which created a Support Fund for Black Audiovisual Professionals in order to offer complementary income to beneficiaries enrolled and selected by their own call (Benfeitoria 2020).

Specific worker groups also benefit from the actions taken by associations and collectives. The Brazilian Composers Union (UBC), in conjunction with Spotify, is offering a monthly fee of R$ 400 (US$ 71.2) to composers and artists affiliated with the institution who declare themselves financially vulnerable due to the pandemic. The funds to cover the amount to be allocated to professionals came from donations made through the crowdfunding platform Benfeitoria and Spotify (Alves, 2020). “Salve a Graxa” (“Save the Grease”) is a movement created in Belo Horizonte with the objective to assist stage assemblers, editors, audio, video and lighting technicians, porters and roadies (the backstage team known as Graxa, “grease,” in the Brazilian music industry). The project is subsidized by donations on the crowdfunding platform Evoé and the amounts collected are being used to purchase food, cleaning and personal hygiene products that will be delivered to these workers (Carlos, 2020).

Finally, the impacts of the pandemic on the cultural sector were evident from the start, since the suspension of concerts and events, the closure or restriction of the operation of bars and cultural venues such as museums, cinemas, art galleries and theaters, were among the first actions to combat the spread of Covid-19. Data from the National Household Sample Survey on Covid-19 (PNAD, carried out by IBGE) indicate that the cultural sector had 1.9 million informal workers in July 2020. An analysis by Ipea, also in July, shows that people who work for themselves were the most affected by the pandemic, having got paid “effectively only 60% of what they regularly get” (Carvalho, 2020). Therefore, the unemployment and wage loss of professionals working in different activities of the cultural sector is a worrying reality that public aid policies, in the context of the pandemic, attempt to mitigate. Although general emergency aid has been “essential for maintaining income among the poorest households” (*id. Ibid.*), again it is still not possible to prove the effectiveness of the measures taken specifically for the cultural scene.

### Results Obtained From the Questionnaire

In the following topics we present the results regarding specific questions about income; access to public and private policies to support the private sector and, finally, adaptations to the virtual environment during periods of social isolation.

#### Income

##### Monthly Income

**FIGURE 1:**
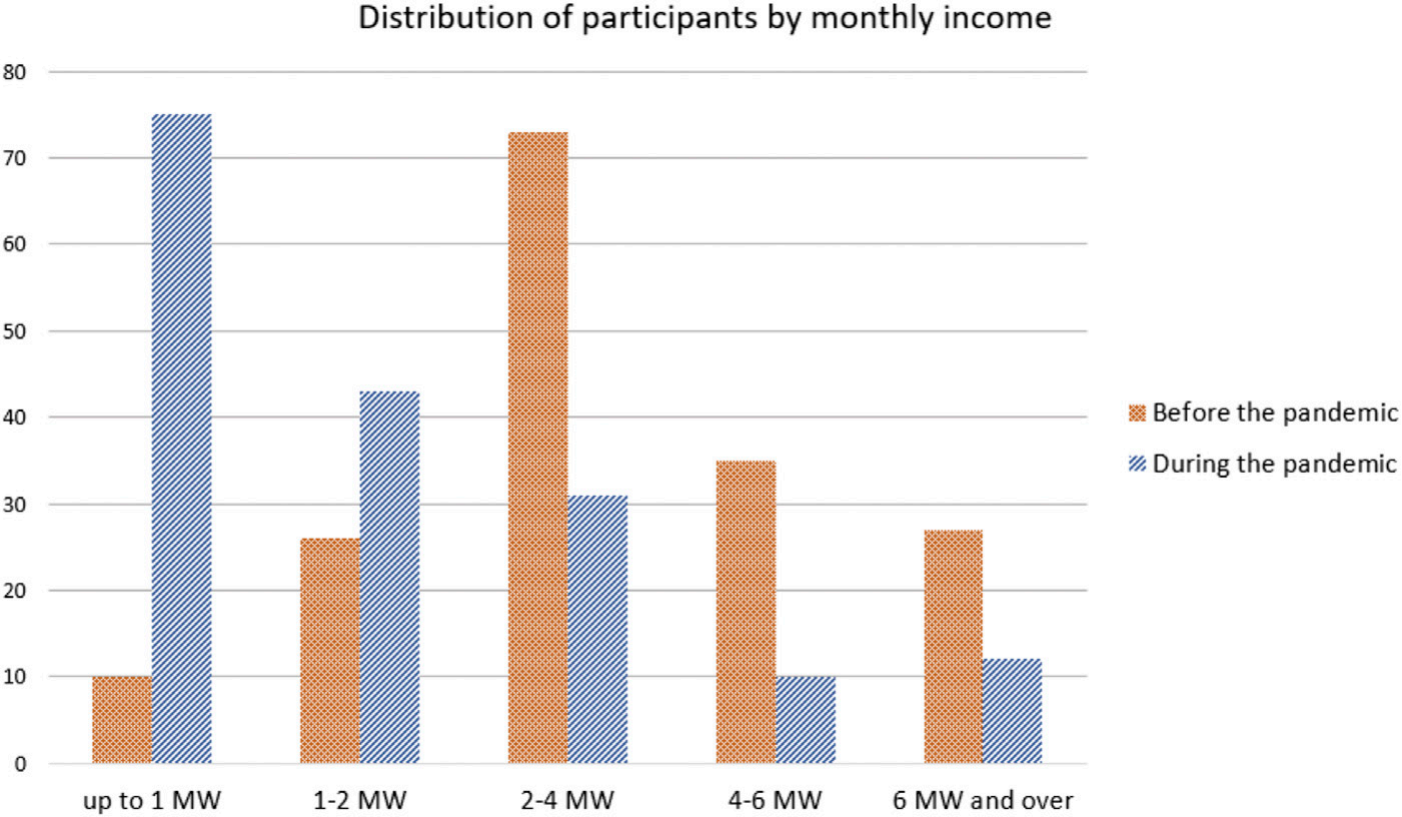
Distribution of participants by monthly income.

The impact of Covid-19 on the financial income of the music sector in Belo Horizonte is profound, considering the average income of musicians before and after the pandemic based on the minimum monthly wage in Brazil, which was–in October 2020—R$1.045 (US$ 186). Figure 1 illustrates the distribution of monthly income among the respondents, calculated by the payroll ranges based on minimum wage and showing a comparison between the pre-epidemic scenario and the status of the current pandemic era. The data depict a significant reduction in the income of music professionals. During this pandemic period, the number of respondents in the lower two minimum wage ranges has increased: those whose income is one minimum wage or less grew from 10 (5.8% of respondents) to 75 people (43.9% of respondents), and those whose income was between 1 and 2 minimum wages increased from 26 (15.2%) to 43 people (25.1%). On the other hand, the three highest minimum wage income ranges took the opposite direction: the number of people with income between 2 and 4 minimum wages dropped from 74 (43.3%) down to 31 people (18.1%); those with income between 4 and 6 minimum wages dropped from 35 (20.5%) to 10 people (5.8%); and, finally, those who were on the top income range of 6 minimum wages and over dropped from 26 (15.2%) to 12 people (7%).

In general, considering all the categories presented here, we can state that in the scenario of the pandemic no respondents have announced an increase in monthly income at the time of the analysis. In Table 1 we describe the effect caused by the pandemic on the monthly income of the respondents, taken as an indication of the monthly wage range before the epidemic. Only 29.2% maintained an income that was similar to the one before the epidemic, while 70.8% showed a decline in pay range. These numbers show a significant reduction in the respondents' income, motivated by factors such as: total or partial disruption of activities related to the music community; the need to adapt professional music services to the online environment which included loss of audience and/or reduction of the amount received for the service; prevention or reduction of other activities unrelated to the music area.

**TABLE 1:** Effect caused by the pandemic on the monthly income of the respondents.

Previous income range	Up to 1 MW	1–2 MW	2–4 MW	4–6 MW	6 MW +
Maintained the same income range	10 (100%)	6 (23.1%)	15 (20.3%)	7 (20%)	12 (46.2%)
Reduction (↓ 1 income range)		20 (76.9%)	25 (33.8%)	11 (31.4%)	3 (11.5%)
Reduction (↓ 2 income ranges)			34 (45.9%)	12 (34.3%)	5 (19.2%)
Reduction (↓ 3 income ranges)				5 (14.3%)	0
Reduction (↓ 4 income ranges)					6 (23.1%)
Total	10	26	74	35	26

We underline the fact that, from all 171 responses, 145 (84.8%) stated that they live on their own income or have people who depend on their income. To illustrate this situation, 77 respondents (45%) mentioned the number of dependents: 35 responses indicate that there is at least one person connected to a musician who also depends on his or her income. 27 respondents said that there are at least two people in this position and, finally, for 15 respondents (about 8% of the total respondents) their income affects themselves and three or more people connected to him or her.

##### Other Sources of Revenue

To a lesser extent, 68 respondents (39% of the 171 respondents) mentioned other non-music services they had to offer during the pandemic to guarantee some sort of payment, ranging from managerial and marketing activities, or freelancing jobs including photography, journalism, home cooking or even working as an app driver.

**FIGURE 2:**
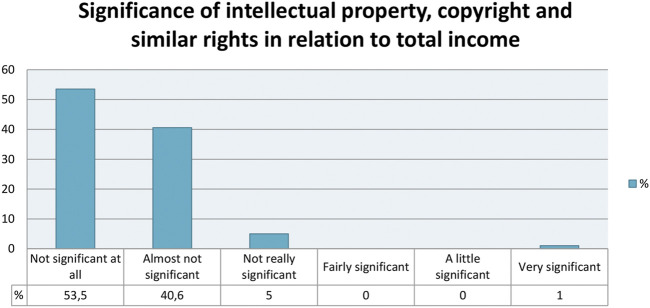
Significance of intellectual property, copyright and similar rights in relation to total income.

But the study also investigates if the respondents had any other source of income besides music-related activities, and its representative share. This section contains 86 responses, which is practically half of the total respondents. We can therefore deduce that the other half of the respondents surveyed depend solely on musical activities–which could explain the many challenges in adapting to the online environment or even adapting to non-music activities as a way to increase income during the pandemic. Going back to the 86 musicians who responded that they have sources of income other than of music production, we have a very balanced picture, in which for 37 respondents (43%) this income represents nothing, a little or very little; while for the remaining 49 (57%) this income is reasonable, important or very important–and again, many of these extra occupations are those mentioned above as being non music related activities that occur frequently during the pandemic, which might be configured as an overlapping indicator in the survey. However, it should also be noted that this section also includes other fixed-income occupations not directly connected to the music service, such as public servants, teachers, retirees, announcers, entrepreneurs, among others. We can notice another characteristic of the survey respondents' universe: the majority do not get income derived from copyright or related rights (Figure 2). The 101 responses here (59% of the total), tell us that income from intellectual property, copyright or related rights, such as image, compositions (CDs, DVDs), teaching material, streaming (YouTube, Spotify, Deezer etc.) or views in social networks represented nothing. For 40 others (40.6%) it represents very little. If we add to these numbers another 70 musicians who skipped the issue because they have no monetization from that direction, we then have confirmation of a strong bottleneck in the virtual medium. We will discuss that in the next topic, in which, it seems, only artists who already have some media visibility can achieve considerable financial return from these strategies.

##### Music Industry: Economically Relevant Activities

The discussion is complemented by the responses of three sections on the following relevant realms: financial, visibility and artistic, as per the list of the activity categories mentioned: “solo musical work ” and/or “in groups”; “Carnival”; “Bars and/or restaurants”; “Celebration and/or religious events”; “Recording studios”; “Multidisciplinary art”; “music teacher”; “music research”; “music centers”; “orchestras”; “popular culture groups.” These three sections were answered by 100% of the respondents, but this is a warning that in many of them there is a tendency to overlap–as, for example, the reference to “solo” or “group” work in “bars and restaurants”—or even an indication of more than one activity as an answer (on average, for each of the three sections, seven answers always indicated that all actions would be important). So, the amount of answers presented here is approximation, more in the sense of stipulating trends and quality guidelines found in each section than in the sense of stipulating quantified exactness.

Therefore, as for the most essential jobs from a financial point of view, it is possible to indicate the great importance of festive events, be they religious or not. At least 38 responses mentioned this specific kind of activity. This was followed closely by working as a music teacher, with at least 33 responses, then, in sequence, performing in bars and restaurants (25) and theaters (20). Solo (20) and group activities (19) are almost normal, followed by Carnival (12), reinforcing the strength of festive events in the sector. With less than 10 explicit occurrences we have the musical corporations, orchestras and popular culture groups.

##### Working in the Music Sector: Activities Relevant to the Visibility of the Professional

It is interesting to compare the results above with the ones in this section, which again considers the same activities listed, but raises the question of what would be the most relevant activities from the point of view of artistic visibility. It is possible to notice many references to performing in groups or solo, which are now equal at the top, with 42 and 35 responses respectively–almost twice the amount of previous mentions. Again, one cannot rule out the overlap of these questions in relation to the other categories (also shown in some responses), but it seems that the importance of visibility of the artistic performance, whether individual or collective, is suggested here. Another interesting datum that emerges from this survey is the importance of bars and restaurants and also of the Carnival of Belo Horizonte as being very relevant for this visibility–for 34 and 29 responses respectively. While we perceive a certain equivalence of bars and restaurants as being prominent places both for the financial aspect (see previous section) and for the visibility of the professional, in this regard Carnival in particular stands out even more. These two categories are far above the other categories previously associated with financial gain, such as ceremonies and events of any kind (19 responses) and recording studios (17). But the greatest incongruity is seen in the activity of music teacher, which had only eight direct responses in terms of visibility, in contrast with the weight of its financial importance as seen previously.

Still, although the answers to the question about music teachers in this section tell us that more than 48% of participants consider classes to be important activities for extra income, only 62% of the corpus answered the question. Again, making a proportional calculation, we have about 30% of respondents indicating the importance of the activity for their income, which leads us to at least two scenarios: either, in fact, few are dedicated to the task of teaching, or the financial reward, albeit relevant, is low.

The questionnaire shows us that, from the point of view of the revenue generated, multidisciplinary art education teams are relevant to about 40% of the respondents. However, given the low adherence to the question, we can imagine that the precariousness/impediment of activities impacts an even smaller number of artists. Only 55.5% of the total respondents proposed to answer it. Proportionally, if we consider the total amount, the impact falls on just over ⅕ of the interviewees.

As for musical research, a perspective that mainly focuses on acting in the academic environment, we can see that this universe means a source of diminished income in the reality of the interviewees. Only 45.6% of the research corpus answered the question, which means modest participation, something already expected given the practical and historical dimension of informality that marks the qualification and professionalization of the area. The already reduced number is under pressure from the response of almost 70% of respondents, who declare that this is an activity that represents nothing in their income composition. Thus, only 30% of respondents to the question say they earn some amount through research grants. Taking the entire corpus, we are talking about less than 12% of respondents. The national situation is helping to hinder this possibility of pay from research, since even before the pandemic, a substantial cut by the federal government in funds and incentives for research in the field of education and the arts had been felt.

##### Working in the Music Sector: Activities Relevant to the Artistic Career

The third section of the survey, which complements the discussion above, asked which activity would be the most relevant from an artistic point of view. Again, we have to point out the same circumstances previously reported for the sections on financial relevance and visibility: the selection of more than one activity per response or even the identification of all as important, as well as the overlapping of responses. Trying to derive some prominence from the set, we detected, as in the question about visibility, that solo activities (41 responses) and group activities (40) stand out, being closely followed by activities in bars and restaurants (37 responses) and various ceremonies and events (34). Then there are the recording studios (23), music teacher and Carnival (17), pointing out a similar degree of convergence between the artistic relevance of this section and the one considered in the previous section regarding the visibility of these activities.

We have already mentioned the Carnival scene, pointing out, for example, its important role for the visibility of the musicians. When we look at the answers to the question that deals with the relationship between professional practices linked to this event and the composition of income, we see the existence of an apparent balance in the composition of the responses. Half of the respondents indicate that the event is very important for their income, while the other half indicates the opposite. However, if we pay attention to the fact that 20% of the respondents skipped this question, we can infer a decrease in the importance of the event for the general composition of the interviewees' income. If we analyze the answers by the respondents who say that indicate Carnival is something that only impacts their income in a reasonable way, we will have the majority saying that Carnival does not significantly affect the composition of income in the annual calendar. It is interesting to see that some time ago Carnival, as was the case of the end of the year festivities, were dates when good fees were paid. This may indicate that the event, in addition to circulating good money, has visibility and has gained new proportions in the capital of Minas Gerais, but has not been able to turn this into significant deliveries from the point of view of income for most musicians. We realized, without a doubt, that the growth of this event, however, created a niche in the music market. It also points to less dependence of musicians on Carnival nowadays, as compared with past conditions. This reveals a possible transformation of the market, which offers a division of labor capable of offering other opportunities for pay, although we still cannot predict the stabilization of this niche. This also happens because we have to wait for the pandemic to unfold so that we can again think about situations that inexorably depend on social contact, such as carnival celebrations.

One piece of data that is presented immediately is that approximately 72% of respondents were significantly impacted in relation to the presentation fees. If we exclude those that indicate that the performances represent only reasonable importance, we still have 58.3% indicating the relevance of the impact. Of the interviewees, 27.7% indicated that they would not withdraw their financial support from there. A little over ¼ of respondents are involved concurrently with other activities, something that mitigates to some extent the negative impact of measures to control the pandemic. It is also possible that the data may reveal a number, which should not be disregarded, of amateur musicians in the composition of the music market.

##### Activity in Bars and Restaurants

**FIGURE 3:**
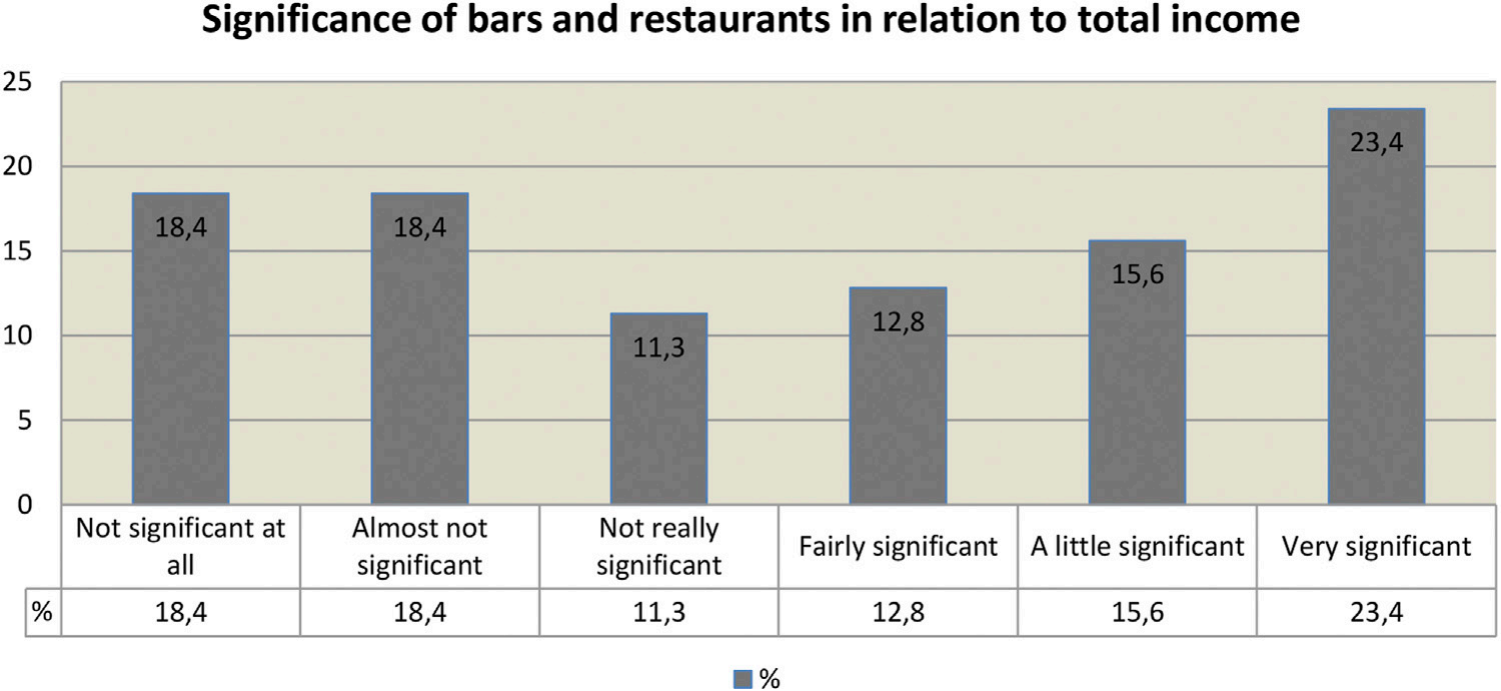
Significance of bars and restaurants in relation to total income.

To continue to understand the impact on the artists’s income, let us now understand the perspective that has implications in bars and restaurants. When analyzing a set of responses by item, it is confirmed that the largest fraction of Figure 3 refers to the importance of bars and restaurants as the musician’s “natural place” (23.4%). However, considering the individual factors, it is important to note that two of the three responses corresponding to the size indicate a small significance of these points. The added numbers, including the responses of those who consider their income from presentations in bars and restaurants as “reasonable,” “significant” or “very significant” to their income, indicate that this source is important for 51.8% of those surveyed, which helped us understand the extent of the damage caused by the need for social isolation.

It is necessary to reinforce that bars and restaurants were closed at the same time as the other commercial establishments, but, on the other hand, they were one of the last to obtain authorization for full reopening, following the security protocol of the committee to fight the pandemic in Belo Horizonte. Even after the operation was authorized, musical performances were still prohibited. Thus, more than half of the musicians continue to make up their income with the good results of presentations in bars and restaurants. It must be said, however, that about 17.5% of the total number of respondents to the questionnaire ignored this question, which drops the consideration of importance to less than half of the researched corpus, reaching 42.7%—a figure that is still quite expressive.

Although in the item above, as we have seen, 51.8% of respondents have given significant relevance to what is earned in performances in these locations, only 39.6% indicate frequent, weekly presentations. If we consider that we have experienced a significant reduction in the number of respondents from the previous question to this, something in the order of 20%, we are faced with data that make us ponder, yet again, on the importance of this practice for the income of those involved in the research. There is, therefore, a possible incongruity in the correlation between the data from the answers to this question and the answers to the previous question, regarding the relationship between income composition and the revenue obtained from presentations in bars and restaurants. There is no expected correspondence between the group that declares the income obtained in bars and restaurants to be important and that which is said to be frequent in presentations in these places. We may need to consider that other, less frequent musicians are able to earn a higher remuneration per performance, which could bring balance to the relationship.

##### Activity at Religious Festivals and Events

**FIGURE 4:**
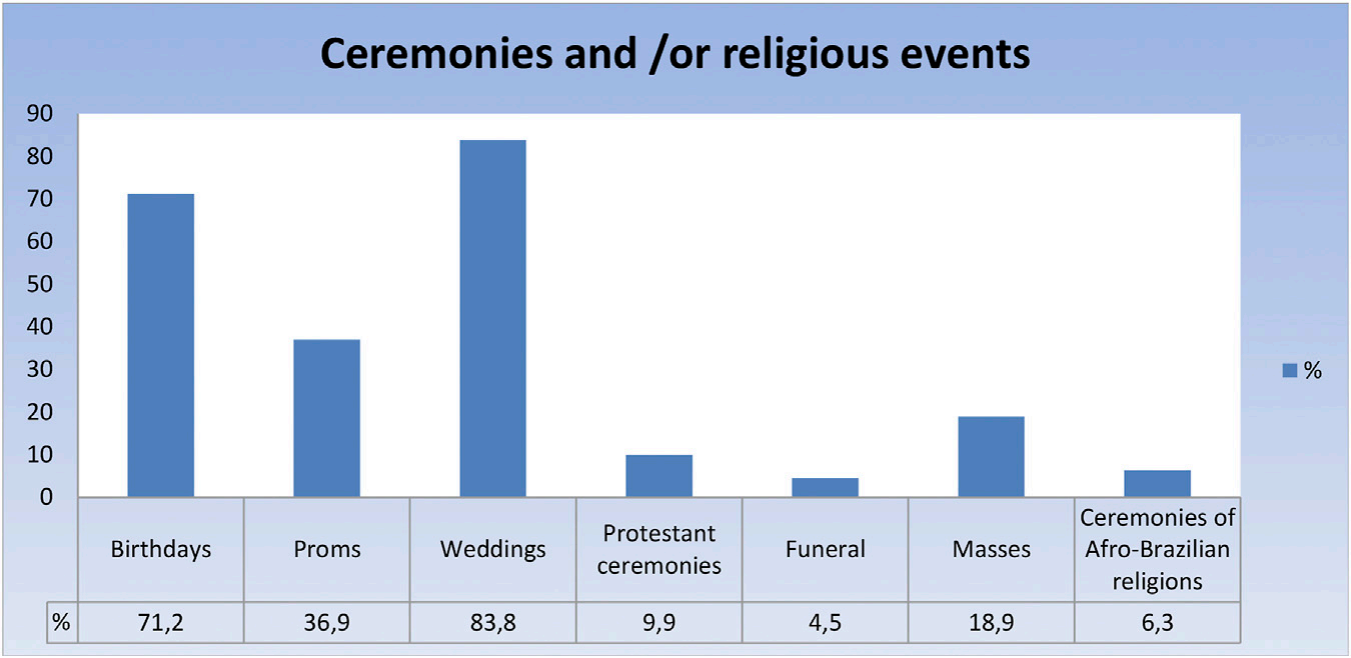
Ceremonies and-or religious events.

When we look at religious ceremonies and events as profitable scenarios, approximately 46% of respondents indicate such events as important opportunities for making money. If we think that 48.1% (a number very close to the 46% mentioned above) of the respondents do not point out bars and restaurants as important sources of remuneration, we can think that ceremonies are occupying an important space of performance, showing themselves as an option to the traditional strongholds of live music. However, such ceremonies were also restricted due to social isolation. Mainly catholic masses, which make up 18.9% of the income generating activities in this category. If we add masses, rituals of African matrices and burials, all impacted either by the ban on the event or by the restriction of the audience present, we arrive at a situation that impacts almost 30% of the respondents. Numbers that could be higher, if we also added the downtime of religious ceremonies. Protestant ceremonies in small spaces have been noticed in the peripheries of the capital. But if we ignore this exceptional condition and include the numbers of the services, we will have 40% of the musicians prevented from earning income in these rituals. Nevertheless, it is the celebratory ceremonies that best describe the impact in this regard. The interruption of birthday parties, proms and weddings produced the greatest impacts because they were an alternative that was already established, even becoming the main element in the composition of income for various musicians (Figure 4). Considering that the question gave the option of marking more than one alternative, we can thus confirm the strong performance of respondents in these areas.

##### Performance in Studios

Leaving the environment of live performances, we proceed to detail the observations of the scenario that involves production and recording procedures. Less than 40% of the interviewees pointed out the activity in recording studios as being of at least reasonable importance in the composition of the income. Therefore, most of them do not have studios as a relevant area of ​​activity, at least in terms of income. The studios are normally responsible in the production chain for the delivery of products with high added value, which makes them an important agent in the professional field of music. In large part, commercial studios have seen their importance mitigated by technological advances and by facilitating access to equipment and resources at reasonable prices. Thus, as previously seen, the offer of home studios has multiplied. Even so, commercial studios continued to play an important role in adapting to the standards required, for example, by laws to encourage culture. The questionnaire shows the maintenance of the importance of commercial studios. It is approximately 5% higher than the incidence of participation of musicians in third party studios when compared to the numbers of home studios. With the reduction in advertising production and the restriction of the operation of commercial establishments, we can imagine the fall in the activity of commercial studios. However, the data that draws the most attention in this item is about the possibilities of the online environment, which even in times of pandemic, when almost everything was transported to the virtual environment, and live performances online became a recurring option–if not income, at least exposure, as will be pointed out later–the respondents did not indicate a relevant occupation of this resource.

##### Performance in Musical Corporations, Popular Culture Groups, Orchestras and Choirs

Still analyzing the composition of income, we present below the topics with low adherence of respondents. They try to measure the income composition of the interviewees through participation in bands, orchestras, choirs, popular culture groups, among other corporations. From this series of questions, the items that address the composition of the recipe through participation in musical corporations and popular culture groups were those that had the highest adherence of respondents, with respectively 36.84 and 38.59% of the total respondents. Taking into account the adhesions to the question about musical corporations, only 12.7% of the respondents indicated some importance of the income earned in this field of activity for the calculation of their revenue. Meanwhile, 77.8% do not see any relevance.

From this group, it remains for us to deal with the responses relating to the participation of respondents in orchestras and choirs. A little over ⅓ of the total respondents answered the question. Of them, only 10.2% indicate that they perceive some relevance of the field in determining their income. In fact, if we move on to the detailed responses, which indicate which orchestras the respondents are active in, we will notice that only 8% of the impacted respondents fall into the activities of this subfield. When it comes to corals, only 31.5% of respondents proposed to answer this item. Of these, 16.8% indicate some relevance of the performance in corals in the composition of income, exceeding by more than 50% the indicators of relevance of participation in orchestras. However, the answer with the greatest relevance remains the one that indicates zero impact on the income. This allows us to realize that the interruption of the activities of orchestras and choirs, aggravated by the need for joint action by several people and, therefore, prolonging downtime, has little impact on the composition of the earnings of the professionals in question–especially if we consider that many of those involved in corporations, orchestras and choirs have some source of stable income established by public or private contracts, which, even in the case of choir members, may even be external to the nature of the musical activity. And another part of choir members also do it voluntarily, establishing a bond that does not involve professional remuneration, but other types of elective affinities.

As it is possible to perceive, we try to listen to the question of income in detail, so that we can arrive at a comprehensive result, capable of revealing as much as possible, and in a more accurate way, the situation of musicians, performers and professionals in the musical field during the pandemic.

##### General Considerations on Income and Social and Racial Inequalities

From the crossing of the respondents monthly income data and their racial identification, before and during the pandemic, some questions stood out: in addition to highlighting the role of structural racism in the income of professionals in the city’s music industry, the data also show that the changes in work relations caused by the pandemic acted to intensify these preexisting racial inequalities. Before the pandemic, respondents who identify as Black (the sum of dark skin and light skin Black people, according to the IBGE criteria) already had a monthly income considerably lower than those who identify as white. In the scenario prior to the pandemic (Table 2), although they represent 58.5% of the survey responses, white professionals are 88.5% among people from the highest income range (6 minimum wages and more); and 65.7% in the second highest income range (between 4 and 6 minimum wages). During the pandemic (Table 3), white professionals now represent 91.7% of the highest income range, and 60% of the second highest.

**TABLE 2:** Monthly income by racial identification - before the pandemic.

Income by racial identification	Up to 1 MW	1–2 MW	2–4 MW	4–6 MW	6 MW +	Total respondents
White	5 (50%)	10 (38.5%)	39 (52.7%)	23 (65.7%)	23 (88.5%)	100 (58.5%)
Black	5 (50%)	12 (46.1%)	34 (46%)	11 (31.4%)	2 (7.7%)	64 (37.4%)
Asian, indigenous or did not answer	0	4 (15.4%)	1 (1.3%)	1 (2.9%)	1 (3.8%)	7 (4.1%)
Total by income range	10	26	74	35	26	171

**TABLE 3:** Monthly income by racial identification - in the context of the pandemic.

Income by racial identification	Up to 1 MW	1–2 MW	2 a 4 SM	4 to 6 SM	6 SM +	Total respondents
White	38 (50.7%)	25 (58.2%)	20 (64.5%)	6 (60%)	11 (91.7%)	100 (58, 5%)
Black	33 (44%)	17 (39.5%)	10 (32.3%)	3 (30%)	1 (8.3%)	64 (37.4%)
Asian, indigenous or did not answer	4 (5.3%)	1 (2.3%)	1 (3.2%)	1 (10%)	0	7 (4.1%)
Total by income range	75	43	31	10	12	171

In the scenario prior to the pandemic (Table 2), Black professionals, although representing only 37.4% of total respondents, were 50% among the lowest income range (up to 1 minimum wage); 46.1% among the second lowest (1-2 minimum wages); 46% among the middle income range (2-4 minimum wages); 31.4% among the second highest (4-6 minimum wages); and only 7.7% of people with higher income (more than 6 minimum wages per month). During the pandemic (Table 3), Black professionals were 44% of the lowest income range; 39.5% of the second lowest, 32.3% of the middle range, only 30% of the second highest income range and 8.3% of the highest one.

Although white professionals have also suffered a significant reduction in their income, the predominance of Black people in the lower income ranges and their considerably lower presence among higher incomes is very outstanding, both in the previous scenario and in the context of the pandemic. Although these data do not apply exclusively to income from activities in the music industry, also including sources of income linked to other parallel professional activities, overall financial stability ends up being a factor of significant relevance for the continuity or abandonment of musical activities, since it directly impacts the ability to invest, study and pursue further qualification in the area.

In this sense, the data analyzed reiterates the need for public and private cultural initiatives to implement affirmative action policies, such as quota systems favoring Black people and other measures of social and racial inclusion during the projects’ execution. Without these actions to promote racial equality, public calls, cultural contests, cultural promotion programs and other types of cultural incentives can perpetuate or intensify preexisting racial inequalities and privileges, considering that white professionals already have a higher monthly income, both previously and during the pandemic, and higher levels of education, which favors access to these and other opportunities in the musical environment.

#### Access to Public Policies

As highlighted in the previous topic, the professionals who answered the questionnaire pointed out a significant decrease in income after the interruption of artistic activities and the closure of cultural spaces since the beginning of the pandemic. This finding, together with the data that will be explored below, collaborates to attest to the importance of public policies to assist professionals in the musical environment in the context of the pandemic.

Income reduction was a reality for 121 respondents, or 71% of the total, given that it can be crossed with the question that addresses access to government financial assistance in the context of the pandemic. Of all the professionals who reported a reduction in income, 66 (54.5%) received government assistance, and most responded that this contribution came from the federal government. However, it must be stressed that this aid did not come from the resources of the Aldir Blanc Law, since the Government of Minas Gerais, responsible for the transfer in the state, is still analyzing the data received from registrations made by professionals and artistic institutions. Presumably, it may be the general emergency aid for informal and self-employed workers of R$ 600 (US $ 107) articulated between the government and the House of Representatives, as previously mentioned.

The registration of people linked to cultural activities in Minas Gerais was initiated on July 30 by the State Secretariat for Culture and Tourism (Secult) for the execution of the emergency aid stipulated by the Aldir Blanc Law. The workers were able to request the contribution, exclusively by online form, until September 25th. In the current phase of the procedure for the money to be effectively passed on to professionals, Secult is verifying the compliance of the 11,320 registrations received in accordance with the rules of the legislation. There is still no stipulated date for the payment of the emergency aid, but in a decree published on October 9, the state government established that the amount of R $ 600 (US $ 107) will be paid for 5 months.

**FIGURE 5:**
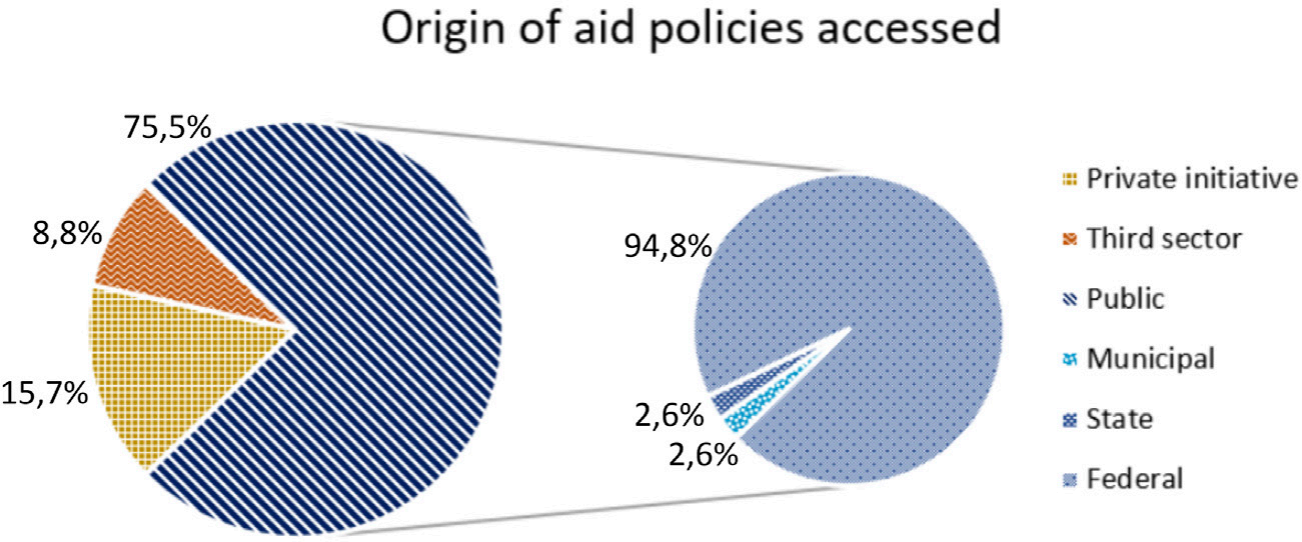
Origin of aid policies accessed.

In general, while 73 respondents (42.7%) said they had received some support or government aid in the context of the pandemic, 16 (9.4%) stated that they had received assistance from the private sector. As mentioned earlier, private sector projects aimed at the cultural sector are also playing an important role in the current scenario, as well as the initiatives of third sector institutions, such as Non-Governmental Organizations (NGOs), unions and associations. Nine people who responded to the questionnaire claimed to have received financial assistance from these institutions during the pandemic period. The following graph (Figure 5) details the data on the access of professionals to public policies in the context of the pandemic, identifying which spheres the aid received comes from.

The data show the importance of public sector actions in assisting workers affected by the crisis intensified by the pandemic, while demonstrating that actions by the private sector and third sector organizations are also possible and relevant.

In the next phase of the research, it will be possible to verify the possible specific difficulties of professionals from the Belo Horizonte culture in accessing such public policies. In any case, it is pertinent to point out some problems in the general allocation of resources, starting with the delay in the approval and execution of legislation aimed specifically at the cultural sector. In addition, there are uncertainties regarding the ability of municipalities to sectorally manage the policies required by the Aldir Blanc Law. Data from the Basic Municipal Information Survey (MUNIC/IBGE) of 2018 show that only 2,352 (42%) of Brazilian municipalities had a culture council and only 654 (9%) constituted their Municipal Culture Plan.

#### Adaptations to the Virtual Environment

**FIGURE 6:**
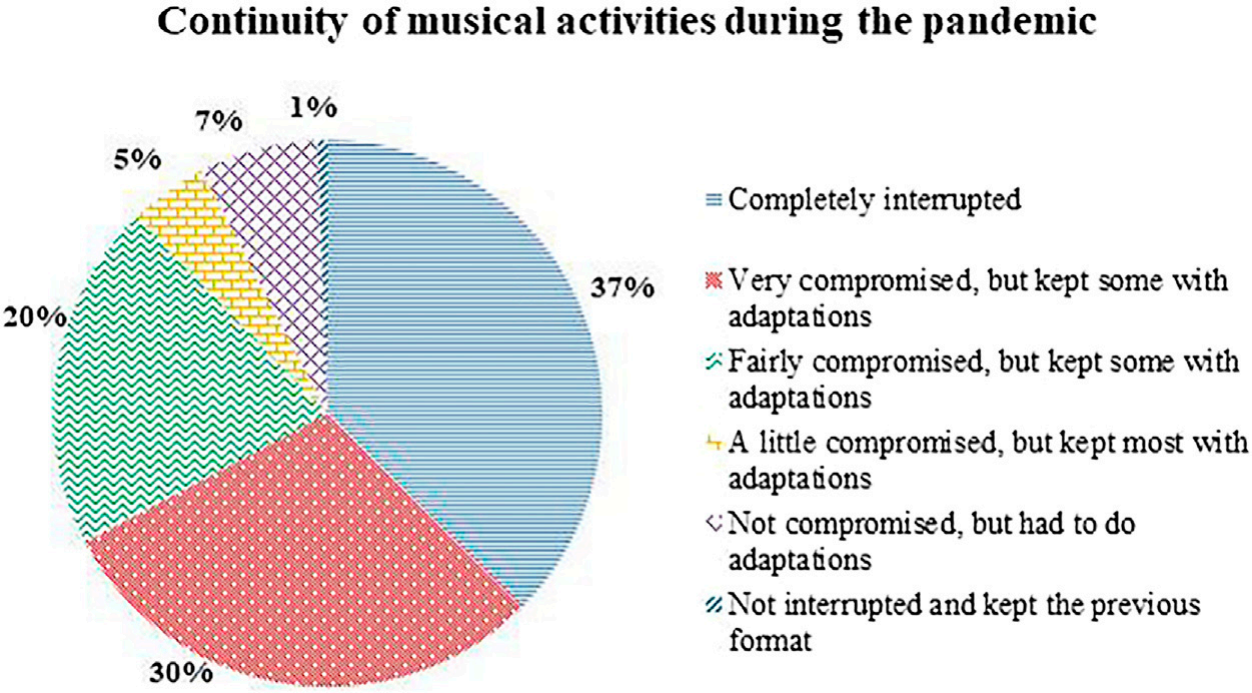
Continuity of musical activities during the pandemic.

Due to the intensely social character of the professional activities of the local musical environment, traditionally involving the physical presence of the public in live presentations of different formats, we seek to understand to what extent the need for isolation and social distancing led to the total or partial interruption of these activities. In Figure 6 we see the impact of social distancing on the continuity/discontinuity of musical activities for respondents. Of the 171 respondents, only one person (0.6%) replied that the activities were not interrupted and could therefore maintain the same format as before the pandemic.

We asked the interviewees to, if they had to make adaptations in their main musical activity due to the context of the pandemic, describe how they made these adaptations, in an open answer field. This question received a total of 105 responses. To present these data, we evaluated the patterns and recurrences that emerged from these responses and grouped them into eight categories of adaptation of professional activities, and the same subject interviewed may have answered more than one of these. The details of these categories of adaptation of activities and the number of respondents who declared them are shown in Table 4.

**TABLE 4:** Categories of adaptations undertaken and number of respondents.

Categories of adaptations undertaken	Number of respondents
Online environment - live performances and open events online (interpretation, production, audio treatment or studio rental for conducting live performances; without financial specification, with voluntary contributions, or without pay)	52
Online environment - online meetings (production, rehearsals, chorus conducting online)	10
Online environment - classes taught or received online (some reported loss of students, increased efforts to search for new students, reducing fees and/or increasing workload to compensate)	40
Face-to-face classes and other work in shared environments (with social distancing, mask use, sanitary protocols)	4
Presentations and private events (face-to-face private events, drive-in shows, creating playlists for private events, online or face-to-face serenades)	9
Recording of own initiative or on demand (remote, online, isolated, or in home studio)	31
Made new investments (in studies, in training, in new musical projects, and/or in equipment)	11
Releasing or selling musical work (selling records remotely, releasing previously recorded content, publishing or disseminating content, songs and/or videos on digital platforms)	10

**FIGURE 7:**
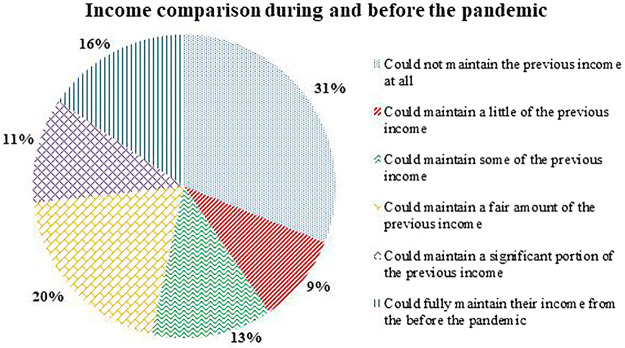
Income comparison during and before the pandemic.

We also asked the interviewees: if they had to make adaptations in their professional musical activities did they maintain the same income as it was before the pandemic? This question had 116 answers, considering that not all respondents needed to make adaptations, many have other sources of income and, among those who had their previous musical activities completely interrupted, there are some that started offering new services, as we can see in Figure 7.

If 105 respondents (61% of the total) detailed which adaptations they made, a very close group of 102 respondents (60%) mentioned their paid musical activities during the pandemic. The main and most obvious of these, the migration to the online environment, had a similar impact on several responses by musicians, especially those who also earn income from education. And so we have an axis of responses that highlights the challenges both of this transition to online classes, as well as to live performances on the web. Common to both: the effort to reformulate the production routine, especially program and lesson strategies; the need to invest in equipment for this production (both sound and ambient lighting, knowledge of software applications, video recording) even if this cost was not covered immediately. On the contrary, in the cases of online classes there was a decrease in students and also in the prices charged–although this also meant, in other statements, new students. Some statements illustrate the challenge: one of them says that “it was necessary to considerably increase the weekly workload, including the need to make an extensive effort to publicize the hours available for online classes.” Another reiterates: “I migrated my classes to online mode and lowered my hourly/classroom value. I got a larger number of students than in the face-to-face mode, and at the same time the workload increased a lot in this online model, due to the production of videos, lesson preparation and virtual teaching materials.” The production and availability of video classes on platforms like YouTube also marked some testimonials.

As seen, despite some setbacks, the practice of music education, important from a financial point of view, was one of the activities that best adapted to the digital universe, and to the resources offered by the internet. Singing teachers, for example, given the impossibility of wearing masks and the imminent risk in the act of singing (given the forceful sprinkling of saliva in the air), even after the beginning of the flexibility, remained somewhat constrained in their teaching activity. However, online platforms have proved to be efficient for conducting virtual classes. This can be seen when 64% of those who answered the question indicate that activities are carried out through digital platforms for interaction and content communication (Youtube, Zoom, Meet, Skype, etc.). These numbers are not more relevant than those that indicate the teaching practice carried out at the teacher’s own residence or at the student’s residence, something that we assume was interrupted during the pandemic.

The pandemic also brought up the issue of live performances online as a possible form of insertion. However, in these responses, many challenges presented above were reiterated: investment in equipment, especially home studio, was a constant point in many responses, not only for the composition and production process for live performances online, but also recording–considering, also, online collaboration and remote multitracks. A respondent even said that he suspended the band’s own artistic activity to rent the studio for live performances online. Others mention meetings and rehearsals online. “I had to find the platform that best suited my type of activity, and learn to use it in the best way. Rehearsals became small individual tutoring sessions.” There were also testimonies that pointed to the focus of production and shipping within online possibilities, or even resorting to the Post Office: “I started to release previously recorded content–videos, singles, EPs of live recordings etc. and thereby created an average volume of content for the year.”

For some testimonies, the proposition of live performances online is important not only as a way of earning income–which sometimes only occurred in the form of a “solidarity cover charge”—but, above all, as a way of staying active before the public, even without financial return. The publication of music or videos also has this objective: to remain in evidence. Some strategies were shared in the responses: “adaptations were made through online parties using meeting platforms (Zoom) and also through live performances on social networks.” Another points out the paid solution for “virtual parties”: “I make playlists for parties and curatorship: I send two new songs via Whatsapp–from my daily music searches–for R$ 5 (one dollar) a month.” And at least three testimonies positively invested in serenades, even online, for events such as birthdays.

Many said that they had to reinvent themselves with the home studio–both performing activities not related to music, and looking for an opportunity of investment–until then never realized–in composition and recording. In a specific case, another problem was mentioned: the quality of the internet connection; and, in another case, also the dismissal from the school where the musician used to teach. Finally, as the questionnaire’s deadline coincided with the beginning of the flexibilization process in Belo Horizonte, some answers already indicated the adequacy to the health protocols (use of mask, hand sanitizer and distancing) both for the classroom environment and for the studios.

## Discussion

We ended the first stage of this research at a time when the municipal administration of Belo Horizonte is already giving in to pressure from different sectors for the reopening of commerce, bars and restaurants. We do not yet know whether Belo Horizonte will sustain the informal title of Capital of Bars in the country after the pandemic. Despite the reopening and recent authorization for live music in these establishments, the ban on agglomerations and the use of masks remains in effect, since the coronavirus is still a public threat that polarizes public opinion between those who minimize the impact of pandemic and those who follow the recommendations of the World Health Organization.

Although part of the musicians have returned to the bars, the public still cannot crowd to listen to the performances. Income, therefore, remains low for the bar musician who depends on a “full house” to survive. The responses to the questionnaires confirm that the income obtained by musicians in bars is significant for most respondents, just as these places stand out in terms of professional visibility and artistic relevance.

Another significant part of these professionals income–religious ceremonies, wedding parties and anniversaries–are not expected to resume soon, although private events and more restricted ceremonies continue to take place. A large part of the respondents had another important source of income from their musical activity in addition to being relatively important as a “showcase” for the musician and as an artistic expression.

Those who embrace music education, although they do not have the weight of dissemination and artistic expression in this activity, as we perceive in the analysis of the responses, consider it extremely significant from a financial point of view–it was this profitable practice that best adapted to isolation conditions, showing an important path of pedagogical performance through digital interconnection resources, nevertheless also being an activity impacted by the pandemic.

Even one of the biggest national events is in danger of not happening in the coming years: Carnival, which is traditionally held between the months of January and March. How can one dance to Carnival without crowding, without people occupying the streets? The impact of the cancellation (or postponement) of Carnival is already happening, with the suspension of the rehearsals of the carnival blocs, which usually generate income for professionals, in the months before the party, and the impossibility of parties held throughout the year, which are animated by musical groups derived from the carnival groups^12^.

But the economic loss will still worsen, since Carnival itself means a moment of great movement for the city. After all, Belo Horizonte has experienced a growth in its carnival in the last decade, reaching the third place among the largest parties in the country. In 2019, there was an audience of 4.3 million people, including 204,000 tourists (Prefeitura De Belo Horizonte, 2020) numbers that converge with the results of this research, which identifies Carnival as an important market niche for musicians and for a productive chain that begins with the organization of events and reaches areas such as food and hospitality.

Although, in general, all professionals have been affected, we find that the impacts of the pandemic show and aggravate preexisting racial inequalities. The responses to the questionnaire showed that Black professionals already had considerably lower income when compared to white professionals, as well as being more severely affected by the reduction in monthly income caused by the pandemic context. In this sense, we highlight the importance of state and private cultural recovery initiatives implementing affirmative actions in their selection processes, in order to repair these structural asymmetries between white and Black professionals.

Another important point is the versatility of these professionals in the face of the adversities in the cultural and musical economy: the majority of the interviewees already had, even before the pandemic, two or more professional activities in the musical environment, and only a minority dedicated themselves to only one activity. This versatility also reveals a certain precariousness of the music market in Belo Horizonte, requiring multiple performances by professionals.

In the context of the pandemic, this versatility was manifested in the diversity of adaptations undertaken by professionals in an attempt to remain active during this period. This set of adaptations ranges from the transfer of activities to remote, isolated, and/or online versions; including the adoption of sanitary measures such as wearing masks, practicing social distancing and frequent hand sanitization for the maintenance and/or gradual restart of some face-to-face activities; up to the creation of new services, releasing content in new formats and investing in new equipment, qualification and/or new artistic projects. However, despite the visible commitment to innovate and adapt to the current scenario, we found that the majority of these professionals were unable to maintain their income equal or similar to the time before the pandemic, reporting loss of customers, reduced demand for services, carrying out unpaid or low paid activities, as well as the need to increase the number of hours worked or resort to activities that are not linked to music, such as driving for personal transport apps.

In addition to the impact for professionals in the music sector, we seek the actions of public authorities–at federal, state and municipal levels–to support the sector during the health crisis and the resulting economic crisis. We realized that some initiatives were carried out in all spheres. However, when confronted with the answers obtained by the questionnaire, we realized that professionals had limited access to these resources. It is worth investigating in the next stage of the research whether those who claim to have had access to federal aid refer to the emergency aid made available to the general population or to specific aid resulting from public policies directed at the sector, in order to understand the difficulties in accessing these measures.

In any case, it is possible to indicate that our results converge with evidence and discussions raised by other research on the impacts of the pandemic on livelihood of music professionals. The approaches are diverse: while some proposals look specifically at musicians, others include them in the broader cultural sector (Dones et al., 2020). Some studies focus on diagnosis (Betzler et al., 2020; Hunt et al., 2020; Crosby and McKenzie, 2021; Spiro et al., 2021); others give more prominence to strategies to survive this scenario and adapt to the future that emerges from there (Tolmie, 2020; Varano, 2020). Although almost all circunscribe research within cities and at most countries, there are those who set out to understand the effects of Covid-19 on musicians from all over a continent (Joffe, 2020). The fact is that this area of ​​professional and artistic activity is especially affected by this health crisis, although it varies depending on the socioeconomic aspects of each population and its local inequalities. In this context, the relevance of public policies to support these workers and follow the changes that the music market has been experiencing has also been frequently highlighted by most of the works.

With that in mind, the application of the methodology developed in this article to the reality of other cities in Brazil or other countries is promising. Within the Brazilian case, for example, another research, in a country level, gets several mainly results similar to our local own, like the “high” or “very high” impact in reducing revenue for 65% of individual artists in Brazil, and also the need of a first financial support, training, services and infrastructure that make it possible to adapt activities to the digital environment (Canedo and Paiva Neto, 2020; Amaral et al., 2020). Other specific surveys on the Brazilian music market also confirmed, among other aspects, the availability and monetization of musicians for online content as a crucial need (DataSim, 2020). By other hand, each location experiences the pandemic in a unique way and may have its specific issues: for example, those Brazilian cities where beach tourism is the main economic resource, even for musicians, and suffer a drop of − 78.9% of Brazilian Tourism revenue, only from March 1st to July 18th, 2020 (Tomé, 2020).

The next stage of this research will be carried out through focus groups, together with people who answered the online questionnaire. We asked, at the end of the questionnaire whose results we present here, if the respondents would be interested in participating in the next phase^13^. The openly formulated question also made room for comments and suggestions to the research team^14^. In this space for open answers, personal reports were elaborated by the respondents regarding the difficulties presented by this time. Among the observations and suggestions about the research, some respondents said they missed more targeted questions about activities related to cultural administration, executive production and technical support and issues related to copyright. There was also a comment pointing out that music research also takes place outside academic circles, contrary to what the form would have suggested.

Such comments and suggestions were recorded and will contribute to the formulation of the script for the next stage of the study, in order to obtain greater depth in what concerns qualitative aspects and in the ways in which this moment of crisis in the musical area materializes in more unique experiences.

## Data Availability

The original contributions presented in the study are included in the article/Supplementary Material, further inquiries can be directed to the corresponding author.

## Footnotes

1 This is the date when the results were first discussed by our research team in order to write the paper and submit it for publishing, soon after the deadline for the survey’s responses. In late April 2021, when the study was reviewed, the World Health Organization (WHO) reported that Brazil reached 381,475 deaths due to Covid-19 and over 14 million confirmed cases. Available at https://covid19.who.int/region/amro/country/br. Accessed April 23, 2021.

2 Numbers collected on October 25, 2020.

3 On January 11, 2021, the commercial activities in Belo Horizonte were brought to “essential only” levels (which only allowed places like supermarkets and pharmacies to open) until February 1, 2021, when the city reopened all services and stores. On March 5th the city put restrictions back in place, with only essential businesses, up until April 22, 2021.

4 This audience is equivalent to 71% of the 120 million Brazilians with internet access. Further information from the Datafolha survey institute also reveals that 91% of the inhabitants of São Paulo, the largest city in the country, have listened to music during the pandemic.

5 All currency conversions noted in this text were made in accordance with the commercial dollar rate signaled by the Central Bank of Brazil on October 23, 2020 (US$ 1 dolar ≅ R$ 5.61 reais).

6 In addition to the researchers who formally integrate the research (as formally registered in Plataforma Brasil), the other researchers from the ESCUTAS group participate as collaborators.

7 See SARTORETO, Felipe. Pesquisa vai dimensionar impactos da pandemia no setor musical - Professora Graziela Mello Vianna, do Departamento de Comunicação Social, falou sobre o estudo em entrevista à Rádio UFMG Educativa [Research will measure pandemic impacts on the music sector - Professor Graziela Mello Vianna, from Social Communication Department, spoke about the study in an interview at Federal University of Minas Gerais Radio Education Channel]. UFMG. September 24th, 2020. Available at https://ufmg.br/comunicacao/noticias/pesquisa-desenvolvida-na-ufmg-quer-dimensionar-impactos-da-pandemia-no-setor-musical [Accessed October 21, 2020].

8 See MATEUS, Bruno. Estudo da UFMG quer entender impactos da pandemia no setor musical em BH [UFMG study wants to understand pandemic impacts on the music sector in BH]. O Tempo. September 15th, 2020. Available at https://www.otempo.com.br/diversao/estudo-da-ufmg-quer-entender-impactos-da-pandemia-no-setor-musical-em-bh-1.2385092. We also gave interviews in the local radios such as Band News, CBN and Itatiaia (not available on line) and finally, in a TV show that was presented after the survey deadline, but also gave a good visibility to the research. See BALANÇO GERAL[TV program]. Pesquisa da UFMG aponta efeitos da pandemia em músicos da Grande BH [UFMG research points out the effects of the pandemic on musicians from Grande BH]. R7. Oct 13th, 2020.Available at https://noticias.r7.com/minas-gerais/balanco-geral-mg/videos/pesquisa-da-ufmg-aponta-efeitos-da-pandemia-em-musicos-da-grande-bh-13102020 [Accessed October 21, 2020].

9 The questionnaire had 174 answers, but three of them were discarded: one, because it was an answer from a person who lived outside the area of ​​this study; the other two were repeated responses from the same respondents, and we considered only the most recent response from each of them as being useful. Therefore, we got 171 valid answers, which will be described in the next section.

10 The complete project (including the survey, the informed consent term, and the questionnaire) and the assessments are available in the Comitée platform (in Portuguese). It can be accessed in https://plataformabrasil.saude.gov.br/visao/publico/indexPublico.jsf;jsessionid=83B5238FE2557C43697DCE195CF35C8D.server-plataformabrasil-srvjpdf130, under the project’s title in Portuguese (“Música em tempos de pandemia: o impacto da Covid-19 no meio artístico”). 1 - click on the middle field “Buscar pesquisas aprovadas” (Search for approval researches) and, once there, fill the field 2 - “Título da Pesquisa” (Research title) with the title mentioned above.

11 “The Programa Bolsa Família (PBF) is a government program introduced in 2003 by the then-president, Lula da Silva. Under PBF low-income families receive cash transfers on the condition that they, for example, send their children to school and ensure they are properly vaccinated. The conditional cash transfer model successfully reduced levels of inequality and hunger–with significantly fewer people living below the poverty line–and closing the historical rural-urban gap.” (Ćirović, 2019).

12 The carnival blocks promote interaction between professional and amateur musicians (during the celebrations, the latter have an opportunity to learn and practice music, especially wind and percussion instruments). It is common for the blocks to have a base band and a leader, who guide the amateurs in the execution of the songs during rehearsals and carnival parades. And it also happens that, throughout the year, the team of professional musicians who acts in a block presents itself as the “Band of that block,” adapting the carnival setlist for situations of balls, parties, festivals and ceremonies (such as weddings, graduations, inaugurations). Therefore, a very relevant music market has been built in the city, surpassing the seasonal character of the party.

13 A group of 70 people volunteered to participate in the focus group (41%), while 24 (14%) preferred not to be in the next phase of these research. 6 respondents (3.5%) answered that they could take part in it depending on how the group would work. In general, questions arose about the time they would spend on the activity.

14 Regarding the comments and suggestions about the research, the presence of thanks and compliments for the initiative was remarkable.

## References

[B1] Aguiar Tito Flávio R. (2018). “Subúrbios e colônias agrícolas: morar e trabalhar nas bordas da cidade,” in Estudos sobre Belo Horizonte e Minas Gerais nos trinta anos do BDMG Cultural. Editors de Freitas Dutra Eliana. Boschi Caio. (Belo Horizonte: BDMG Cultural), 81–98.

[B2] Alex Berlin (2020). Deine Stadt - Dein Programm. Available at: https://www.alex-berlin.de/startseite.html (Accessed May 5, 2020).

[B3] Ali A. Ahmed M. Hassan N. (2020). Socioeconomic Impact of COVID ‐19 Pandemic: Evidence from Rural Mountain Community in Pakistan. J. Public Aff., e2355. 10.1002/pa.2355 32904946 PMC7460993

[B4] Alves Soraia. (2020). “Spotify e UBC lançam fundo para artistas durante crise da Covid-19.” Brainstorm9. Available at: https://bit.ly/3xnjRho (Accessed April 25, 2021).

[B5] Amaral Denise. Meira. (2020). Shows virtuais catapultam brasileiros à lista de artistas mais assistidos no mundo, São Paulo: Folha de S. Paulo Available at: https://www1.folha.uol.com.br/ilustrada/2020/07/shows-virtuais-catapultam-brasileiros-a-lista-de-artistas-mais-assistidos-do-mundo.shtml.

[B95] Amaral R. C. Franco P. A. I. Lira A. L. G. (2020). Pesquisa de percepção dos impactos da COVID-19 nos setores cultural e criativo do Brasil, Paris/Brasília:Organização das Nações Unidas para a Educação, a Ciência e a Cultura-UNESCO Available at: https://datastudio.google.com/u/0/reporting/88bf6daa-3f58-4f5a-bb3f-9d4f5c3dc73b/page/FdCXB?s=gUJpgJdXnvQ (Accessed May 26, 2020)

[B6] Americans for the Arts (2020a). Coronavirus Impact Survey. Available at: https://surveys.americansforthearts.org/s3/CoronavirusImpactSurvey (Accessed May 7, 2020).

[B7] Americans for the Arts (2020b). The Impact of COVID-19 on Artists and Creative Workers. Available at: https://www.surveygizmo.com/s3/5532991/6539d78e3593 (Accessed May 7, 2020).

[B8] Amormino Luciana. (2020). BH 120 anos: temporalidades e memória em narrativas jornalísticas sobre o aniversário da cidade. Anais do XIX Encontro Anual da Compós XIX, 1–21.

[B9] Art Power H. K. (2020). “ART Power HK: Celebrating the Arts in Hong Kong. Available at: https://www.artpowerhk.com/en/about (Accessed May 6, 2020).

[B10] Bahia Claudio. L. M. (2004). JK: política, arte e arquitetura - uma experiência modernista. Cadernos de Arquitetura e Urbanismo 11 (12), 119–137.

[B11] Bahia Denise. Marques. (2011). “A arquitetura política e cultural do tempo histórico na modernização de Belo Horizonte (1940-1945).” PhD diss. Belo Horizonte: Federal University of Minas Gerais.

[B12] Barreto Abílio. (1944). O vertiginoso evoluir de Belo Horizonte. Revista Belo Horizonte 166, 43.

[B13] Becker H. (1993). Métodos de pesquisa em ciências sociais. São Paulo: Hucitec.

[B14] Benfeitoria(2020). Fundo de Amparo aos Profissionais Do Audiovisual Negro para ações emergenciais durante a pandemia de COVID-19. Available at: https://bit.ly/3gBQ8eG (Accessed April 25, 2021).

[B15] Berlin - Senate Department for Culture (2020). Cultural Policy. Available at: https://www.berlin.de/sen/kultur/en/cultural-policy/ (Accessed May 5, 2020).

[B16] Berlin - United We Stream (2020). Streams. Available at: https://unitedwestream.berlin/stream/ (Accessed May 5, 2020).

[B17] Berlin (a)live (2020). Was Ist Berlin (A)live? Available at: https://www.berlinalive.de/was-ist-berlin-alive (Accessed May 5, 2020).

[B18] Berliner Philarmoniker Digital Concert Hall (2020). How it Works. Available at: https://www.digitalconcerthall.com/en/info (Accessed May 5, 2020).

[B19] Bernard H. R. (2005). Research Methods in Anthropology: Qualitative and Quantitative Approaches. Lanham, MD: AltaMira Press.

[B20] Betzler D. Loots E. Prokůpek M. Marques L. Grafenauer P. (2020). COVID-19 and the Arts and Cultural Sectors: Investigating Countries' Contextual Factors and Early Policy Measures. Int. J. Cult. Pol., 1–19. 10.1080/10286632.2020.1842383

[B21] Biernarcki P. Waldorf D. (1981). Snowball Sampling-Problems and Techniques of Chain Referral Sampling. Sociological Methods Res. 10 (2), 141–163.

[B22] Boston Singers Resource (2020). Application for Funds: COVID-19 Emergency Relief. Available at: https://www.bostonsingersresource.org/programs/bsrf/application (Accessed May 7, 2020).

[B23] Brandão C. A. (2018). “Belo Horizonte entre palavras e formas: o que restou da modernidade?,” in Estudos sobre Belo Horizonte e Minas Gerais nos trinta anos do BDMG CulturalEliana de Freitas Dutra and Caio Boschi, 21–40. (Belo Horizonte: BDMG Cultural).

[B24] Buehnen Berlin. (2020). Schedule. Available at: https://www.berlin-buehnen.de/en/schedule/ (Accessed May 5, 2020).

[B25] Buzatti Lucas. (2020). Conectando a cidade a partir da Cultura. Belo Horizonte: O Tempo Available at: https://bit.ly/3dChiQZ (Accessed April 20, 2021).

[B26] Campos L. M. (2007). A música e os músicos como problema sociológico. rccs 78, 71–94. 10.4000/rccs.756

[B27] Canedo D. P. Paiva Neto C. B. (2020). *Pesquisa Impactos da Covid-19 na Economia Criativa: relatório final de pesquisa*. Salvador: Observatório da Economia Criativa (OBEC); Santo Amaro: UFRB. Available at: https://ufrb.edu.br/proext/images/pesquisa_covid19/RELAT%C3%93RIO_FINAL_Impactos_da_Covid-19_na_Economia_Criativa_-_OBEC-BA.pdf.

[B28] Carlos Helvécio. (2020). Projeto Salve a Graxa reforça campanha de doações. Estado de Minas. Available at: https://bit.ly/2QQZoAV.

[B29] Carrol Rory. (2020). “Most Americans to Avoid Sports, Other Live Events before Coronavirus Vaccine: Reuters/Ipsos.” *Reuters* . Available at: https://www.reuters.com/article/us-health-coronavirus-usa-events/most-americans-to-avoid-sports-other-live-events-before-coronavirus-vaccine-reuters-ipsos- idUSKCN22A2AK.

[B30] Carvalho S. S.(2020). Os efeitos da pandemia sobre os rendimentos Do trabalho e o impacto Do auxílio emergencial: os resultados dos microdados da PNAD Covid-19 de agosto. Carta de Conjuntura - Ipea, 6.Available at: https://bit.ly/2TlKxg0 (Accessed October 23, 2020).

[B31] Chacham V. (1994). “A memória dos lugares em um tempo de demolições: a Rua da Bahia e o Bar do Ponto na Belo Horizonte das décadas de 30 e 40.” Master’s thesis. Belo Horizonte: Federal University of Minas Gerais.

[B32] Ćirović S. (2019). Bolsa Família in Brazil.” Centre for Public Impact. Available at: https://bit.ly/3murewS (Accessed December 16, 2020).

[B33] Cités et Gouvernements Locaux Unis(CGLU). (2020). “#CULTUREcovid19 La mobilisation culturelle des villes et des gouvernements locaux dans la crise du COVID-19.” Available at: https://www.uclg.org/fr/media/nouvelles/culturecovid19-la-mobilisation-culturelle-des-villes-et-des-gouvernements-locaux.(Accessed May 5, 2020)

[B34] Carroll K. (2020b). Arts and Culture Departments. Available at: https://www.boston.gov/departments/arts-and-culture (Accessed May 7, 2020).

[B35] Carroll K. (2020a). Boston Establishes Artist Relief Fund in Response to Coronavirus. Available at: https://www.boston.gov/news/boston-establishes-artist-relief-fund-response-coronavirus?fbclid=IwAR266pSEATAOVAkjanntwOG-NhHHT7lSkdaJAESfiRVOl2cI2Tv2udp_w04%C2%A0 (Accessed April 26, 2021).

[B36] Coep (2021). Bioética. Available at: https://www.ufmg.br/bioetica/coep/ (Accessed April 23, 2021).

[B37] Conep (2021). Comissão Nacional de Ética em Pesquisa. Available at: http://conselho.saude.gov.br/comissoes-cns/conep/ (Accessed April 23, 2021).

[B38] Costa Débora. Veríssimo. (2019). Moderno e eterno: sobre os discursos de modernidade em Belo Horizonte. Patrimônio e Memória 15 (2), 215–230. Available at: http://pem.assis.unesp.br/index.php/pem/article/view/843.

[B39] Cristaldo Heloisa. (2020). Câmara dá prioridade para mãe chefe de família no auxílio emergencial. Agência Brasil. Available at: https://bit.ly/2QUxAf8.

[B40] Crosby P. McKenzie J. (2021). Survey Evidence on the Impact of COVID-19 on Australian Musicians. SSRN J. 10.2139/ssrn.3707325

[B41] Cultural I. (2020). Confira os selecionados no edital de emergência Arte como Respiro. Available at: https://bit.ly/3xqhvhI (Accessed April 25, 2021).

[B42] Datasim (2020). COVID-19 - Impacto no mercado da música Do Brasil. Available at: https://datasim.info/wp-content/uploads/2020/04/Pesquisa-DATA-SIM-Covid-19-Brasil.pdf?utm_source=mailchimp&utm_campaign=0300c4c2e1f0&utm_medium=page (Accessed April 27, 2021).

[B43] Denora T. (2000). Music in Everyday Life. Cambridge: Cambridge University Press. 10.1017/cbo9780511489433

[B44] Dias F. C. (1975). “Gênese e expressão grupal Do Modernismo em Minas.,” in Affonso Ávila. Editors Modernismo O., and edited by (São Paulo: Perspectiva), 165–178.

[B45] Dolabela M. (1987). ABZ Do Rock Brasileiro. São Paulo: Estrela do Sul.

[B46] Dolabela M. (1997). “Cronologia e discografia da MPB em Belo Horizonte.” *Varia Historia* 18. DH/FAFICH/UFMG.

[B47] Dones M. Pascual F. Martín S. Pérez J. (2020). Efectos de la crisis del COVID‐19 sobre el mercado laboral de la ciudad de Madrid (abril del 2020). Madrid: Colegio de Economistas de Madrid y Asociación Centro de Predicción Económica.

[B48] Góes G. Athias L. Martins F. Silva F. (2020). O setor cultural na pandemia: O teletrabalho e a Lei Aldir Blanc. Carta de Conjuntura - Ipea 6. Available at: https://bit.ly/2HwUW5w.

[B49] Grasse J. (2020). Milton Nascimento and Lô Borges's - the Corner Club. New York: Bloomsbury Academic. 10.5040/9781501346866

[B50] Groves R. M. Floyd Fowler J. Couper M. P. Lepkowski J. M. Singer E. Tourangeau R. (2009). Survey Methodology. Hoboken, NJ: Wiley-Interscience.

[B51] Habermas J. (1984). The Theory of Communicative Action: Reason and the Rationalization of Society. Boston: Beacon Press.

[B52] Hanage W. Qiu X. Kennedy-Shaffer L. (2020). Snowball Sampling Study Design for Serosurveys in the Early COVID-19 Pandemic. Available at: https://nrs.harvard.edu/URN-3:HUL.INSTREPOS:37363145. 10.1093/aje/kwab098 PMC8083564 33831177

[B53] Hong Kong Arts Development Council (2020). Support Scheme for Arts and Cultural Section. Available at: https://www.hkadc.org.hk/en/grants-and-scholarship/grants/support-scheme-for-arts-cultural-sector (Accessed April 24, 2021).

[B54] Hunt M. Gedgaudas L. Seman M. (2020). “Initial Impacts of the COVID-19 Crisis on the Music Industry in Colorado and the Denver Metropolitan Region.” Report. *Colorado Creative Industries, Denver Arts And Venues And Regional Economic Development Institute* . Available at: https://bit.ly/3sP3VkD (Accessed October 19, 2020).

[B55] Joffe A. (2020). Covid-19 and the African Cultural Economy: an Opportunity to Reimagine and Reinvigorate? Cult. Trends. 10.1080/09548963.2020.1857211

[B56] Krueguer R. Casey M. A. (2014). Focus Groups: A Practical Guide for Applied Research. Thousand Oaks, CA: SAGE.

[B57] Kugel S. (2007). A Town where All the World Is a Bar. New York Times. Available at: https://www.nytimes.com/2007/10/28/travel/28next.html (Accessed October 21, 2020).

[B58] Kugel S. (2014). “36 Hours in Belo Horizonte, Brasil”. New York Times. Available at: https://www.nytimes.com/2014/04/27/travel/36-hours-in-belo-horizonte-brazil.html (Accessed October 21, 2020).

[B59] Kulturleben Berlin (2020). Kulturleben Berlin. Available at: https://english.kulturleben-berlin.de/?noredirect=en_US (Accessed May 5, 2020).

[B60] Lisboa. (2020). Covid-19 medidas e informações. Available at: https://www.lisboa.pt/covid-19-medidas-e-informacoes/a-cidade/cultura%20acessado%20em%2018/05/2020 (Accessed May 5, 2020).

[B61] Machado Ana. Flávia. Freire Débora. Cavalcante Rodrigo. Vaz de Melo Gabriel. Demattos Alice. (2020). “Nota técnica: Efeitos da Covid-19 na Economia da Cultura no Brasil. Cedeplar”. Cedeplar/UFMG. Available at: https://cedeplar.ufmg.br/noticias/1235-nota-tecnica-efeitos-da-covid-19-na-economia-da-cultura-no-brasil (Accessed October 21, 2020).

[B62] Maciel Victor. (20202020). “Um em cada quatro municípios de Minas Gerais ainda não solicitou os recursos da Lei Aldir Blanc”. Ministério Do Turismo. Available at: https://bit.ly/2J6P041 (Accessed October 23, 2020). 10.29388/978-65-81417-09-3-0

[B63] Martins Bruno. V. (2009). Som imaginário – A reinvenção da cidade nas canções Do Clube da Esquina. Belo Horizonte: UFMG.

[B64] Medeiros J. M. D. Machado L. R. d. S. (2015). O potencial musical de Belo Horizonte como motor de uma estratégia de desenvolvimento local. Per Musi (31), 258–283. 10.1590/permusi2015a3114

[B65] Montevideo (2020). Plano de apoio. Available at: https://montevideo.gub.uy/sites/default/files/biblioteca/comunicadodepartamentodecultura.pdf (Accessed May 8, 2020).

[B66] Montevideo-Cultura (2020b). Bibliotecas Itinerantes: Llévate Un Libro a Casa. Intendencia Montevideo. Available at: https://montevideo.gub.uy/noticias/cultura/llevate-un-libro-a-casa (Accessed May 8, 2020).

[B67] Montevideo-Cultura (2020a). Montevideo-cultura. Available at: https://montevideo.gub.uy/noticias/cultura (Accessed May 8, 2020).

[B68] Morgan David. L. (1993). Successful Focus Groups: Advancing the State of the Art. Newbury Park, CA: SAGE. 10.4135/9781483349008

[B69] Musicovid Network (2020). An International Research Network. Available at: https://www.aesthetics.mpg.de/en/research/department-of-music/musicovid-an-international-research-network.html (Accessed April 27, 2021).

[B70] Noceti Javier. (2020). El plan de apoyo a la cultura por el que la IM destina 10 millones de pesos a los artistas. Montevideo: Montevideo Portal Available at: https://www.montevideo.com.uy/Noticias/El-plan-de-apoyo-a-la-cultura-por-el-que-la-IM-destina-10-millones-de-pesos-a-los-artistas-uc751453 (Accessed May 8, 2020).

[B71] NYC Cultural Affairs (2020). DCLA Covid-19 Coronavirus Resources. Available at: https://www1.nyc.gov/site/dcla/resources/coronavirus-dcla.page#COVID (Accessed May 7, 2020).

[B72] Paula J. A. (2020). *Minas Gerais - visão de conjunto e perspectivas*. Belo Horizonte: Scriptum. Available at: https://issuu.com/assembleia.mg/docs/minas_gerais_joa_o_pdf_para_issuu (Accessed April 22, 2021).

[B73] Paula J. A. (2018). “Belo Horizonte - Quais Horizontes?” In Estudos sobre Belo Horizonte e Minas Gerais nos trinta anos do BDMG Cultural, Belo Horizonte: BDMG Cultural, 171–179.

[B74] Peoples Cultural Plan (2020b). An Urgent List of Demands for Arts and Culture in the Face of NYC State of Emergency. Hyperallergic. Available at: https://hyperallergic.com/548145/nyc-state-of-emergency-covid-19/ (Accessed May 7, 2020).

[B75] Peoples Cultural Plan (2020a). Homepage. Available at: https://www.peoplesculturalplan.org/(Accessed May 7, 2020).

[B76] Prefeitura de Belo Horizonte (2020). Carnaval de Belo Horizonte cresce em 2019 e encanta 4,3 milhões de foliões. Available at: https://prefeitura.pbh.gov.br/noticias/carnaval-de-belo-horizonte-cresce-em-2019-e-encanta-43-milhoes-de-folioes (Accessed October 21, 2020).

[B77] Santos M. P. A. D. Nery J. S. Goes E. F. Silva A. D. Santos A. B. S. D. Batista L. E. (2020). População negra e Covid-19: reflexões sobre racismo e saúde. Estud. Av. 34 (99), 225–244. 10.1590/s0103-4014.2020.3499.014

[B78] Small C. (1998). Musiking: The Meaning of Performing and Listening. London: Wesleyan UP.

[B79] Sound Diplomacy (2020). Music Cities - Resilience Handbook. Available at: https://www.sounddiplomacy.com/better-music-cities (Accessed October 19, 2020).

[B80] Spiro N. Perkins R. Kaye S. Tymoszuk U. Mason-Bertrand A. Cossette I. (2021). The Effects of COVID-19 Lockdown 1.0 on Working Patterns, Income, and Wellbeing Among Performing Arts Professionals in the United Kingdom (April-June 2020). Front. Psychol. 11 (February), 1–17. 10.3389/fpsyg.2020.594086 PMC7902701 33643111

[B81] Teixeira N. (1994). Beto Guedes faz inventário de sucessos. Jornal Hoje em Dia. 10.1016/b978-0-08-100596-5.02993-0

[B82] Teixeira N. (2008). Diversidades convergentes: subsídios para modelo de sistema de informação em incubadoras artístico-culturais a partir de estudo comparado entre Brasil e Canadá. PhD diss. Universidade Federal de Minas Gerais Available at: https://repositorio.ufmg.br/handle/1843/ECID-7NXHKX.

[B83] Teixeira N. (1996). Sepultura Com Cor “Brasilis”. Jornal Hoje em Dia, March 19, 1996.

[B84] Teixeira N. (1999). Um rock no meio Do caminho: subsídios para a implantação de um sistema de informação artístico-cultural em Belo Horizonte. Master's thesis. Universidade Federal de Minas Gerais Available at: https://repositorio.ufmg.br/handle/1843/BUOS-92XG2Z.

[B85] Tolmie D. (2020). 2050 and beyond: A Futurist Perspective on Musicians' Livelihoods. Music Education Res. 22 (5), 596–610. 10.1080/14613808.2020.1841133

[B86] Tomé L. M. (2020). Setor de turismo - impactos da pandemia. Caderno setorial Etene 5 (August), 1–1248. Available at: https://www.bnb.gov.br/s482-dspace/handle/123456789/300.

[B87] Universidade Federal de Minas Gerais (2020). UFMG apoiará produções culturais sobre a pandemia; inscrições abertas até o dia 26. Belo Horizonte: Diretoria de Ação Cultural-UFMG.

[B88] Varano J. I. (2020). Estrategias y desafíos de la industria musical en tiempos pandemia y virtualidad. Question/Cuestión 1 (May), e306–14. 10.24215/16696581e306

[B89] Vilela P. R. (2020). Caixa detalha calendário de pagamentos Do auxílio emergencial extensão. Agência Brasil, September 29, 2020. Available at: https://bit.ly/3sywEtJ.

[B90] Ville de Paris (2020). Un plan de soutien à destination des entreprises et des associations. Available at: https://bit.ly/3xwqa2m (Accessed May 20, 2020).

[B91] Vinuto J. (2014). A amostragem em bola de neve na pesquisa qualitativa. Temat. 22 (44), 203–220. 10.20396/tematicas.v22i44.10977

[B92] Weber M. (1998). Sociologie de la musique. Paris: Édition Métailié.

[B93] Werneck H. (1992). O desatino da rapaziada: jornalistas e escritores em Minas Gerais. São Paulo: Companhia das Letras.

[B94] Wisnik J. M. (1989). O som e o sentido: uma outra história da música. São Paulo: Companhia das Letras.

