# Supplementary material for: Covid-19 Impact on the Music Sector in Belo Horizonte (Minas Gerais, Brazil)
Source: Front Sociol. 2021 Jun 29;6:643344. doi: 10.3389/fsoc.2021.643344 (PMC8276977; doi:10.3389/fsoc.2021.643344)
Supplement: Supplementary file 1 [file DataSheet1.pdf]

Presentation and Consent Form

FREE AND CLARIFIED CONSENT TERM (FCCT)

You are being invited to participate, voluntarily, in the research “Music in times of pandemic: the impact of Covid-19 on the artistic sector”. Our goal is to map the difficulties faced by professionals in the musical scenario in Belo Horizonte and the Metropolitan Region in the context of the Covid-19 pandemic, also seeking to learn about the measures undertaken by the professionals themselves and by public and private entities dealing with these issues.

Your contribution has a lot of importance to our research. Before deciding whether or not to participate in the survey, we ask you to read the information below.

The survey includes questions about your professional performance in the music industry, the relevance of this work in your professional life, and your access to financial support initiatives. The estimated response time is approximately 10 minutes. The collected data will be used only for this research. At any time, whether before, during or after the survey, you can request further clarification, refuse to participate, or withdraw from participation. In any of these cases, you will not be harmed or held responsible in any way. If you feel uncomfortable and want to give up the participation, just close the survey without sending it, or request the deletion of the answer you sent. In case of doubts or other request related to this research, you can contact the researchers in charge, Professor Graziela Mello Vianna and Professor Nísio Teixeira, by e-mails grazielamv@ufmg.br and nisiotei@ufmg.br.

We believe that the results will help us to understand your point of view on the impacts of the pandemic on the creative economy and, at the end of the research, this diagnosis will support proposals for solid actions for the recovery of the sector. The research results will be published in the form of scientific papers, such as articles, books, or presentations at events. In the survey, we will ask for your email address to send a copy of this Informed Consent Form and a copy of your answers. Your identification will be visible only to our team of researchers and will be kept confidential. Thus, your name and e-mail will not be disclosed, published, or shared with other people.

Other important research information

Justification for conducting the research: The great paradox in the COVID-19 time is that “the public is consuming art, music and cultural goods in an accelerated pace, but not contributing financially enough to ensure that artists, musicians, and other professionals of the creative economy, whose work they are consuming, can meet their own basic needs. For the vast majority, their art is not paying their bills, nor [even] the second and sometimes the third job they need to have, just to maintain their capability to create ”, cites the recently released Musical Resilience Manual of Cities, available at #BetterMusicCities by experts from Sounddiplomacy.com, who are linked to the music economy for urban development. The social distance caused by the pandemic highlights the importance of music for people's mental health, even though we are temporarily prevented from sharing in the same physical space of the musical experience. With the closure of bars, nightclubs, concert halls and other spaces for musical performances, the on-site modality of these activities was suspended indefinitely. Daily during the pandemic, artists put available recorded videos or make broadcast live (called “lives”) free of charge. Among these, only

a minority obtains some sort of payment, either from public or private initiatives or through the monetization of platforms.

Research goals: We seek to understand the impact of the pandemic on the Belo Horizonte musical scenario and to highlight the visibility and survival strategies of some of its social actors. Based on the data collected through the survey and the levy of emergency financial contributions from public and private initiatives, it will be make a diagnosis of the scenario in Belo Horizonte and the Metropolitan Region, as a pilot project for possible subsequent application in other Brazilian cities.

Research population: Professionals in the musical production chain performing in Belo Horizonte and the Metropolitan Region.

Procedures to which they will be submitted: The collected data will be grouped and compared for elaborating a diagnosis of the pandemic impacts on the musical scenery of Belo Horizonte and the Metropolitan Region, to be published in scientific works, without disclosing the participants identities.

Risks in taken part in the survey: Some questions can cause discomfort or embarrassment to the participants, such as items on payment and financial difficulties. If this happens, you can pause the filling and return to it later, or withdraw participating, without any penalty

Benefits of participating in the research: At the end of the research, you will have contributed to the scientific knowledge about the impacts of the pandemic on the musical scenery in Belo Horizonte and will have expressed your point of view as a professional in the sector, your difficulties and demands in this scenario. Our expectation is that the research can contribute to support the elaboration of public policies for the sector in the short to medium term, starting from the creation of a public document gathering the results to foment the cultural debate with the municipal council management.

Access to final or partial results of the research: At the end of completing the survey, the participant will receive by email a copy of their own responses. The results will be published as scientific works and in the form of a public document, to which we will give wide publicizing.

Costs involved in participating in the survey: You will not have any sort of expense to participate in the survey and you will not receive any financial compensation for taken part in it.

The research was approved by the UFMG Research Ethics Committee (COEP-UFMG), having Professor Graziela Mello Vianna and Professor Nísio Teixeira as responsible researchers. In case of doubts or questions about the research, you can contact us by e-mails grazielamv@ufmg.br and nisiotei@ufmg.br or by phone (31) 3409-5012 (temporarily unavailable due to the COVID-19 pandemic. During this period, if needed, you can contact directly the responsible professor for the research by calling 31998749007).

In case of doubts regarding the ethical aspects of the research, you can contact the Research Ethics Committee (COEP-UFMG): Antônio Carlos, 6627 Avenue - Administrative Unit II, 2nd floor, Campus Pampulha - UFMG. Telephone: 3409-4592 / E-mail: coep@prpq.ufmg.br.

*Obrigatório *Mandatory

1. Endereço de e-mail * * e-mail address

2. Do you agree to participate in the survey? *

Marcar apenas uma oval. (Mark only one option)

- Yes, I do.
- No, I do not. Thank you.

Pular para a pergunta 3 Skip to question 3

Questionnaire

In this section, we will ask some questions about you and your professional performance in the musical sector.

3. What is your age group? *

Marcar apenas uma oval. (Mark only one option)

- 18-30
- 31-40
- 41-50
- 51 or more

4. What is your gender? *

Marcar apenas uma oval. (Mark only one option)

- Female
- Male
- Non-binary
- Rather not answer

5. What is your educational background? *

Marcar apenas uma oval. *(Mark only one option)*

- Unfinished Secondary School
- Finished Secondary School
- Unfinished University Level
- Finished University Level
- Unfinished Post-Graduation
- Finished Post-Graduation

6. What is your racial identification? (According to features used by IBGE - (Brazilian Institute of Geography and Statistics) *

Marcar apenas uma oval. *(Mark only one option)*

- White
- Black (dark skin Black)
- 'Brown' color (light skin Black)
- 'Yellow' color
- Indigenous color
- Rather not answer

7. Which musical sector do you act? (It can be checked more than one alternative) *

Marque todas que se aplicam. *(check all that apply)*

- [ ] Composition
- [ ] Interpreting (voice)
- [ ] Interpreting (instrument)
- [ ] Arrangement
- [ ] Regency
- [ ] Production (executive)
- [ ] Production (musical)
- [ ] Artistic Direction
- [ ] Technical Area
- [ ] Teaching
- [ ] Research
- [ ] DJ
- [ ] Luthier (craftsperson)

8. If you checked "Interpreting (instrument), specify which one (s):

9. If you checked "Technical Area", specify which function (s) :

10. What is your living location? *

Marcar apenas uma oval. *(Mark only one option)*

- ( ) "Barreiro"
- ( ) south-Center
- ( ) East
- ( ) North-East
- ( ) North-West
- ( ) North
- ( ) West
- ( ) "Pampulha"
- ( ) "Venda Nova"
- ( ) BH Metropolitan Region
- ( ) Outro: ____________________

11. Which is the main region of your professional acting in Belo Horizonte? *

Marque todas que se aplicam. *(check all that apply)*

- [ ] "Barreiro"
- [ ] South-Center
- [ ] East
- [ ] North-East
- [ ] North-West
- [ ] North
- [ ] West
- [ ] "Pampulha"
- [ ] "Venda Nova"
- [ ] BH Metropolitan Region
- [ ] Online Sites

Pular para a pergunta 12 *Skip to question 12*

Insertion in the music scenery of the city

Considering your insertion in the city's musical scenario, which of the activities listed below would you fit into? You can answer more than one alternative if necessary, or skip the question when you do not carry out the professional practice in question.

For activities that apply to your professional performance, we will ask you to specify what proportion it represents in your income (on a scale from 0 to 5, in which: 0 = represents nothing; 1 = represents very little; 2 = represents little; 3 = represents reasonably; 4 = represents significantly; 5 = represents very significantly).

12. Solo and / or group musical performance: if you deal with this activity, specify what proportion it represents in your income. If not, skip the question.

Marcar apenas uma oval. *(Mark only one option)*

- ( ) 0 - represents nothing
- ( ) 1 - represents very little
- ( ) 2 - represents little
- ( ) 3 - represents reasonably
- ( ) 4 - represents significantly
- ( ) 5 - represents very significantly

13. If you perform musically solo and / or in a group, specify which one (s), and in which musical genre (s). If not, skip the question.

14. Carnival performance: if you deal with this activity, specify what proportion it represents in your income. If not, skip the question.

Marcar apenas uma oval. *(Mark only one option)*

- 0 - represents nothing
- 1 - represents very little
- 2 - represents little
- 3 - represents reasonably
- 4 - represents significantly
- 5 - represents very significantly

15. Performance in bars and / or restaurants: if you deal with this activity, specify what proportion it represents in your income. If not, skip the question.

Marcar apenas uma oval. *(Mark only one option)*

- 0 - represents nothing
- 1 - represents very little
- 2 - represents little
- 3 - represents reasonably
- 4 - represents significantily
- 5 - represents very significantly

16. If you perform in bars and restaurants, specify the frequency. If not, skip the question

Marcar apenas uma oval. *(Mark only one option)*

- Fixed weekly performance (1 or 2 times per week)
- Fixed weekly performance (3 or more times per week)
- Fixed monthly performance (1 or 2 times per month)
- Fixed monthly performance (3 or more times per month)
- Sporadic performance

17. Performance in ceremonies and / or religious events: if you deal with this activity, specify what proportion it represents in your income. If not, skip the question.

Marcar apenas uma oval. *(Mark only one option)*

- 0 - represents nothing
- 1 - represents very little
- 2 - represents little
- 3 - represents reasonably
- 4 - represents significantly
- 5 - represents very significantly

18. If you perform at ceremonies and / or religious events, specify which one (s). Check more than one alternative if necessary, or add another alternative in the "other" option. If not, skip the question.

Marque todas que se aplicam. *(check all that apply)*

- [ ] Birthday parties
- [ ] Proms
- [ ] Wedding
- [ ] Religious Cult
- [ ] Funerals
- [ ] Mass
- [ ] "Terreiros" (African matrices religion sites)

Outro: [ ] ______________________

19. Acting in recording studios: if you deal with this activity, specify what proportion it represents in your income. If not, skip the question.

Marcar apenas uma oval. *(Mark only one option)*

- 0 - represents nothing
- 1 - represents very little
- 2 - represents little
- 3 - represents reasonably
- 4 - represents significantily
- 5 - representa muito significantly

20. If you work in recording studios, specify which one (s). If not, skip the question.

Marque todas que se aplicam. *(check all that apply)*

- [ ] Home studio or own studio
- [ ] Third part commercial studio
- [ ] Online sites

21. Acting in multidisciplinary art education teams: if you deal with this activity, specify what proportion it represents in your income. If not, skip the question.

Marcar apenas uma oval. *(Mark only one option)*

- 0 - represents nothing
- 1 - represents very little
- 2 - represents little
- 3 - represents reasonably
- 4 - represents significantly
- 5 - represents very significantly

22. Working as a music teacher: if you deal with this activity, specify what proportion it represents in your income. If not, skip the question.

Marcar apenas uma oval. *(Mark only one option)*

- 0 - represents nothing
- 1 - represents very little
- 2 - represents little
- 3 - represents reasonbly
- 4 - represents significantly
- 5 - represents very significantly

23. If you are a music teacher, specify where (check more than one alternative if necessary). If not, skip the question.

Marque todas que se aplicam. *(check all that apply)*

- [ ] Private music school
- [ ] Public municipal music school
- [ ] Public state music school
- [ ] Federal public music school
- [ ] Private lessons at your own home
- [ ] Private lessons at the student's home
- [ ] Lessons in public elementary schools
- [ ] Lessons in private fundamental schools
- [ ] Lessons in public secondary schools
- [ ] Lessons in private secondary schools
- [ ] Lessons in public institutions of higher education
- [ ] Lessons in private institutions of higher education
- [ ] Video lessons via internet on youtube
- [ ] Video lessons via internet at own platform
- [ ] Lessons online (google meet, zoom, skype)

24. Engaging in musical research: if you deal with this activity, specify what proportion it represents in your income. If not, skip the question.

Marcar apenas uma oval. (Mark only one option)

- 0 - represents nothing
- 1 - represents very little
- 2 - represents a little
- 3 - represents reasonably
- 4 - represents significantly
- 5 - represents very significantly

25. If you are engaged in music research, please specify in which context. If not, skip the question.

Marcar apenas uma oval.(Mark only one option)

- Scientific initiation with scholarship
- Scientific initiation without scholarship
- Graduation with scholarship
- Graduation without scholarship
- Master with scholarship
- Master without scholarship
- Doctorate with scholarship
- Doctorate without scholarship
- Post-Doctorate with scholarship
- Post-Doctorate without scholarship

26. Acting in musical corporations (martial bands, for example): if you deal with this activity, specify what proportion it represents in your income. If not, skip the question.

Marcar apenas uma oval. (Mark only one option)

- 0 - represents nothing
- 1 - represents very little
- 2 - represents little
- 3 - represents reasonably
- 4 - represents significantly
- 5 - represents very significantly

27. If you are acting in musical corporations, specify which one (s). If not, skip the question

28. Acting in an orchestra (philharmonic, chamber, etc.): if you deal with his activity, specify what proportion it represents in your income. If not, skip the question.

Marcar apenas uma oval. (Mark only one option)

- 0 - represents nothing
- 1 - represents very little
- 2 - represents little
- 3 - represents reasonably
- 4 - represents significantly
- 5 - represents very significantly

29. If you are acting in orchestra, specify which one (s). If not, skip the question.

30. Acting in choir: if you deal with this activity, specify what proportion it represents in your income. If not, skip the question.

Marcar apenas uma oval. (Mark only one option)

- 0 - represents nothing
- 1 - represents very little
- 2 - represents little
- 3 - represents reasonably
- 4 - represents significantly
- 5 - represents very significantly

31. If you are acting in a choir, specify which one (s). If not, skip the question.

32. Acting in popular culture groups: if you deal with this activity, specify what proportion it represents in your income. If not, skip the question.

Marcar apenas uma oval. (Mark only one option)

- 0 - represents nothing
- 1 - represents very little
- 2 - represents little
- 3 - represents reasonably
- 4 - represents significantly
- 5 - represents very significantly

33. If you are acting in popular culture groups, specify which one (s). If not, skip the question.

34. Among the activities mentioned [solo and / or group musical performance; Carnival; bars and / or restaurants; ceremonial and / or religious events; recording studios; multidisciplinary art education teams; music teacher; musical research; musical corporations; orchestras; choirs; popular culture groups], which is the most relevant in your career from an artistic point of view? *

35. Among the activities mentioned [solo and / or group musical performance; Carnival; bars and / or restaurants; ceremonial and / or religious events; recording studios; multidisciplinary art education teams; music teacher; musical research; musical corporations; orchestras; choirs; popular culture groups], which is the most relevant from the point of view of artistic visibility? *

36. Among the activities mentioned [solo and / or group musical performance; Carnival; bars and / or restaurants; ceremonial and / or religious events; recording studios; multidisciplinary art education teams; music teacher; musical research; musical corporations; orchestras; choirs; popular culture groups], which is the most relevant from a financial point of view? *

37. Do you have another source of income besides the activities related to music? If so, in what area or sector? If not, skip the question.

38. If you have another income besides the activities related to music, specify what is the proportion that it represents in your total income. If not, skip the question.

Marcar apenas uma oval. *(Mark only one option)*

- 0 - represents nothing
- 1 - represents very little (up to 25%)
- 2 - represents little (from 25% to 50%)
- 3 - represents reasonably (from 50% to 75%)
- 4 - represents significantly (from 75% to 99%)
- 5 - represents very significantly (100%)

39. Do you have income from intellectual property, copyrights or related rights, such as image, compositions (CDs, DVDs), teaching material, streaming (YouTube, Spotify, Deezer, etc.), views on social networks? If so, how much do they represent in your average monthly income? If not, skip the question.

Marcar apenas uma oval. *(Mark only one option)*

- 0 - represents nothing
- 1 - represents very little (up to 25%)
- 2 - represents little (from 25% to 50%)
- 3 - represents reasonably (from 50% to 75%)
- 4 - represents significantly (from 75% to 99%)
- 5 - represents very significantly (100%)

40. Have you done any other paid activities not related to musical scenario during the pandemic time? If so, which one (s)? If not, skip the question.

41. Have you done any paid musical activity during pandemic time? If so, which one (s)? If not, skip the question.

42. Use the scale below to tell if your main musical activity has been stopped altogether or if you have found any way to adapt to this new context. *

Marcar apenas uma oval. *(Mark only one option)*

- 0 - it has been stopped completely
- 1 - it was quite compromised, and I kept very little with adaptations
- 2 - it was reasonably compromised, and I kept part of it with adaptations
- 3 - it was a little compromised, and I kept most of it with adaptations
- 4 - it was not compromised, but I need to make adaptations
- 5 - it has not been stopped, and I kept the same format

43. If you needed to make adaptations to your main musical activity due to the pandemic, describe how you made these adaptations. If not, skip the question.

44. Do you live on your own income or have dependents on it? *

Marcar apenas uma oval. *(Mark only one option)*

- Yes
- No

45. If you have dependents on your income, specify how many people besides yourself.

Marcar apenas uma oval. *(Mark only one option)*

- 1
- 2
- 3 or more

46. If you needed to make adjustments to your main musical activity due to the pandemic, specify whether that activity maintained the same income as before the pandemic. If you have not made any adjustments, skip the question.

Marcar apenas uma oval. (Mark only one option)

- 0 - no, anyway
- 1 - very little
- 2 - little
- 3 - reasonably
- 4 - significantly
- 5 - completely

47. Considering the current minimum salary (MS) in Brazil (R $ 1,045.00), what was your average income before the pandemic? *

Marcar apenas uma oval. (Mark only one option)

- up to 1 MS
- from 1 to 2 MS
- from 2 to 4 MS
- from 4 to 6 MS
- over 6 MS

48. Again, considering the current minimum salary (MS) in Brazil (R $ 1,045.00), what is your current income in the context of the pandemic? *

Marcar apenas uma oval. (Mark only one option)

- up to 1 MS
- from 1 to 2 MS
- from 2 to 4 MS
- from 4 to 6 MS
- over 6 MS

Pular para a pergunta 49 Skip to question 49

Access to public and private financial support initiatives for citizens and / or musical sector

The Ministry of Health classified the whole country as community transmission, in addition to the need to qualify the information on the circulation of the Coronavirus in Minas Gerais, from 03/23/2020. In this section, we will ask questions about your access to public and private financial support initiatives before that date and after that one (during the context of the pandemic).

These initiatives can be in the form of direct payments to citizens or in the form of financial support to the musical sector, such as promotion notices, cultural incentive projects, cultural contests, awards and similar.

49. Before the pandemic, had you received any financial support or assistance from the government? *

Marcar apenas uma oval. *(Mark only one option)*

- ◯ Yes
- ◯ No

50. If you had received financial support or assistance from the government before the pandemic, please specify which one (s). Check more than one alternative if necessary. If not, skip the question.

Marque todas que se aplicam. *(check all that apply)*

- ☐ Municipal Council (City Hall)
- ☐ State (Minas Gerais State Government)
- ☐ Federal
- ☐ International

51. After 03/23/2020, in the context of the pandemic, did you receive any financial support or assistance from the government? *

Marcar apenas uma oval. *(Mark only one option)*

- ◯ Yes
- ◯ No

52. If you received any financial support or assistance from the government in the context of the pandemic, please specify which one (s). Check more than one alternative if necessary. If not, skip the question.

Marque todas que se aplicam. *(check all that apply)*

- [ ] Municipal Council (City Hall)
- [ ] State (Minas Gerais State Government)
- [ ] Federal
- [ ] International

53. Before the pandemic, had you received any financial support or assistance from the private sector? *

Marcar apenas uma oval. *(Mark only one option)*

- ( ) Yes
- ( ) No

54. In the context of the pandemic, did you receive any financial support or assistance from the private sector? *

Marcar apenas uma oval. *(Mark only one option)*

- ( ) Yes
- ( ) No

55. Before the pandemic, had you received any financial support or assistance from the third sector, such as Non-Governmental Organizations (NGOs), unions, associations, religious movements or similar? *

Marcar apenas uma oval. *(Mark only one option)*

- ( ) Yes
- ( ) No

56. In the context of the pandemic, had you received any financial support or assistance from the third sector, such as Non-Governmental Organizations (NGOs), unions, associations, religious movements or similar? *

Marcar apenas uma oval. *(Mark only one option)*

- yes
- No

57. Use the space in this last question to say whether you are interested in participating in the second phase of the survey, as a focus group format. If you want, you can also leave any comments or suggestions to the researchers.

Este conteúdo não foi criado nem aprovado pelo Google.

Google Formulários

This content was not created or approved by Google

Google Forms
